# Exploring Ln(III)-Ion-Based Luminescent Species as Down-Shifters for Photovoltaic Solar Cells

**DOI:** 10.3390/ma16145068

**Published:** 2023-07-18

**Authors:** Gabriela Brito-Santos, Cecilio Hernández-Rodríguez, Beatriz Gil-Hernández, Joaquín Sanchiz, Inocencio R. Martín, Benjamín González-Díaz, Ricardo Guerrero-Lemus

**Affiliations:** 1 Departamento de Química, Facultad de Ciencias, Universidad de La Laguna (ULL), Avenida Astrofísico Francisco Sánchez S/N, 38206 La Laguna, Tenerife, Spain; gbritosa@ull.edu.es; 2 Departamento de Física, Facultad de Ciencias, Instituto Universitario de Materiales y Nanotecnología, Universidad de La Laguna (ULL), Avenida Astrofísico Francisco Sánchez S/N, 38206 La Laguna, Tenerife, Spain; imartin@ull.edu.es (I.R.M.); rglemus@ull.edu.es (R.G.-L.); 3 Departamento de Química, Facultad de Ciencias, Instituto Universitario de Materiales y Nanotecnología, Universidad de La Laguna (ULL), Avenida Astrofísico Francisco Sánchez S/N, 38206 La Laguna, Tenerife, Spain; beagher@ull.edu.es (B.G.-H.); jsanchiz@ull.edu.es (J.S.); 4 Departamento de Ingeniería Industrial, Escuela Superior de Ingeniería y Tecnología, Universidad de La Laguna, Camino San Francisco de Paula S/N, 38206 La Laguna, Tenerife, Spain; bgdiaz@ull.edu.es

**Keywords:** luminescent, down-shifting, solar energy, external quantum efficiency, lanthanide ions

## Abstract

In this work, we have compiled our research on lanthanide-based luminescent materials for use as down-shifter layers in photovoltaic (PV) mini-modules. The complexes we have prepared (C1–17), with formulas [Eu_2_(phen)_2_(bz)_6_] (C1), [Eu_2_(bphen)_2_(bz)_6_] (C2), [Eu(tta)_3_bphen] (C3), [Eu(bta)_3_pyz-phen] (C4), [Eu(tta)_3_pyz-phen] (C5), [Eu(bta)_3_me-phen] (C6), [Er(bta)_3_me-phen] (C7), [Yb(bta)_3_me-phen] (C8), [Gd(bta)_3_me-phen] (C9), [Yb(bta)_3_pyz-phen] (C10), [Er(tta)_3_pyz-phen] (C11), [Eu_2_(bz)_4_(tta)_2_(phen)_2_] (C12), [Gd_2_(bz)_4_(tta)_2_(phen)_2_] (C13), [EuTb(bz)_4_(tta)_2_(phen)_2_] (C14), [EuGd(bz)_4_(tta)_2_(phen)_2_] (C15), [Eu_1.2_Gd_0.8_(bz)_4_(tta)_2_(phen)_2_] (C16), and [Eu_1.6_Gd_0.4_(bz)_4_(tta)_2_(phen)_2_] (C17), can be grouped into three families based on their composition: Complexes C1–6 were synthesized using Eu^3+^ ions and phenanthroline derivatives as the neutral ligands and fluorinated β-diketonates as the anionic ligands. Complexes C7–11 were prepared with ligands similar to those of complexes C1–6 but were synthesized with Er^3+^, Yb^3+^, or Gd^3+^ ions. Complexes C12–17 have the general formula [M1M2(bz)_4_(tta)_2_(phen)_2_], where M1 and M2 can be Eu^3+^, Gd^3+^, or Tb^3+^ ions, and the ligands were benzoate (bz^–^), 2-thenoyltrifluoroacetone (tta^–^), and 1,10–phenanthroline (phen). Most of the complexes were characterized using X-ray techniques, and their photoluminescent properties were studied. We then assessed the impact of complexes in the C1–6 and C12–17 series on the EQE of PV mini-modules and examined the durability of one of the complexes (C6) in a climate chamber when embedded in PMMA and EVA films. This study emphasizes the methodology employed and the key findings, including enhanced mini-module efficiency. Additionally, we present promising results on the application of complex C6 in a bifacial solar cell.

## 1. Introduction

Lanthanides are a group of 15 metallic elements in the periodic table. According to the selection rule, f−f transitions are Laporte forbidden, so the trivalent lanthanide ions (Ln^3+^) are highly stable when incorporated into a host lattice (ligands). The surrounding crystal field developed by the host material destroys the spherical symmetry of the electronic structure, promoting the transition of an electron from a lower-energy f orbital to a higher-energy f orbital. This process is called an induced electric dipole (ED) transition [1] and gives rise to optical absorption and emission. Three types of transitions are possible when the lanthanide ion is under the influence of a ligand field [2]: f−f transitions, the transfer of a 4f electron into a 5d subshell, sometimes called hypersensitive or pseudo-quadrupolar transitions because they seem to follow the selection rules of electric quadrupolar (EQ) transitions, genuine electric quadrupolar transitions, and charge-transfer (CT) transitions (metal to ligand or ligand to metal).

A significant advancement in lanthanide chemistry occurred with the introduction of cryptand ligands, which effectively enclose the lanthanide ion, safeguarding it against solvent molecules that dampen its emission [3]. When the arms of the cryptand incorporate light-harvesting units, they function as an antenna, capturing light and transmitting the energy to the lanthanide ion. The creation of antenna systems centered around lanthanides, which enable the collection and conversion of light to different frequencies, holds significant implications for the advancement of photonic devices and sensors. Lanthanides (III) have unique optical properties that make them potentially useful in areas such as lighting [4], biological imaging [5], and solar energy conversion [6], where the wavelength-converting materials can be incorporated into either the solar cell architecture or a luminescent solar concentrator [7].

Despite their poor spectral response in short-wavelength regions of the solar spectrum (mainly due to thermalization losses from the absorption of high-energy photons [8], the high reflective index, and shallow penetration depth of UV-blue irradiation (e.g., less than 100 nm for a wavelength of 400 nm) [9]), solar cells based on, for example, Si, GaAs, CIGS, or CdTe can still be improved. The most promising approaches to improving the short-wavelength response are the use of luminescent down-shifting (DS) and down-conversion (DC) layers [10,11]. DS is a process where one low-energy photon is created by one high-energy photon absorbed (one-to-one photon process) [12]. DC is a one-to-two photon process, where a high-energy photon is split into two lower-energy photons. Quantum cutting [13] is one approach to DC because a single photon with higher energy can be split into two photons with a similar wavelength, and the theoretical quantum efficiency is above 100% [14]. However, no experimental proof has yet been reported to demonstrate an efficiency gain using DC materials at the top of a c-Si cell. Theoretical studies predict a maximum conversion efficiency of around 40% [15,16] due to the low absorption cross-section of the down-converters.

**Figure 1:**
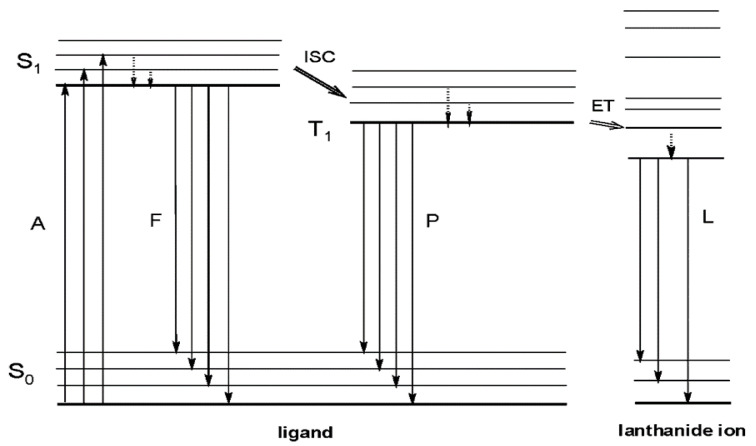
Schematic representation of photophysical processes in lanthanide (III) complexes (antenna effect). Abbreviations: A = absorption; F = fluorescence; P = phosphorescence; L = lanthanide-centered luminescence; ISC = intersystem crossing; ET = energy transfer; S = singlet; T_1_ = triplet. Full vertical lines indicate radiative transitions; dotted vertical lines indicate non-radiative transitions. Reprinted with permission from [19]. Copyright 2009 American Chemical Society.

Despite not exceeding 100% efficiency, down-shifting can be used to improve PV efficiency. By shifting the photons with shorter wavelengths to lower energy, they can be absorbed more effectively by solar cells. Rare-earth organic complexes are an important class of DS materials for PV modules [10]. Unlike organic dyes, fluorophores [17], or semiconductor quantum dots (QD) [18], lanthanide complexes are stable, have large Stokes shifts, and avoid self-absorption losses, where the luminescence process occurs in three steps (Figure 1) [19].

The photophysical process for emission begins with the absorption of light by appropriate ligands, which undergo a transition from their fundamental singlet state (S_0_) to an excited singlet state (S_1_). Efficient intra-energy conversion from the singlet (S_1_) to the triplet (T_1_) states of the ligand occurs through non-radiative intersystem crossing (ISC), followed by resonant energy transference (ET) from the T_1_ state of the ligand to the excited state of the Ln^3+^ ion, which transitions to one of its emissive levels [20,21]. The excited singlet state can be deactivated radiatively to the ground state (molecular fluorescence), and the triplet state T_1_ can be deactivated radiatively to the ground state, by the spin-forbidden transition T_1_ → S_0_ (molecular phosphorescence). The energies of the S_1_ singlet and T_1_ triplet states of the ligands that we have used in this work are shown in Table S1 of the Supplementary Material (Table S1). In the case of fluorine-containing diketonates, recent reviews can be mentioned [22,23].

Our group has been working on luminescent DS rare-earth-based complexes in recent years. Complexes C1–6 [24,25,26,27,28,29,30,31] use ternary europium (III) complexes embedded in poly(methyl methacrylate) (PMMA), while complexes C12–17 [32] are mixed-ligand bimetallic complexes prepared with the ions Eu^3+^, Gd^3+^, and Tb^3+^ embedded in ethylene-vinyl acetate (EVA) as a down-shifting material for silicon-based solar cells. Complexes C7–11 [33] were also prepared with other lanthanide (III) ions, and preliminary studies on their photophysical processes involved in photoluminescence were conducted.

Several methods have been documented in the literature for integrating LDS layers into solar structures. These include spin-on coating techniques [34], deposition of lanthanide-based DS layers on c-Si PV using ethylene-vinyl acetate (EVA) [35], incorporation of Eu(III) or different luminescent organic dyes in the encapsulants [36,37,38,39], re-optimization of the AR-coating [40], implementation of planar luminescent DS layers [41], utilization of CASN:Eu^2+^ in PMMA [42], and inclusion of the DS layer in the encapsulation process [43], among others. Although it is not the subject of this work, we want to mention that lanthanide ions are also involved in up-conversion processes [44], in which emission occurs at a higher energy than the excitation (anti-Stokes phenomenon).

External quantum efficiency (EQE) serves as a measure to assess the improvement achieved by employing DS layers in solar cells. EQE is defined as the ratio of the number of electrons produced by the device to the number of incident photons at each wavelength. This parameter helps evaluate the losses that contribute to the reduction in the measured short circuit current density (J_SC_) from the maximum achievable photocurrent [45].

Integrating lanthanide down-shifting layers into solar cells poses several challenges that must be addressed, including cost-effectiveness, stability, and efficiency [46,47]. As solar energy continues to grow in importance as a renewable energy source, advancements in down-shifting will play an increasingly crucial role in addressing these challenges [48,49,50,51,52,53,54,55,56,57,58,59,60,61,62,63,64].

Among various PV solar cell technologies, conventional c-Si p–n junction solar cells have been dominating the solar market during recent decades. Several new techniques have been used to enhance PV solar cell performance and efficiency, with the use of selective emitters, local back surface fields, or an interdigitated back contact structure in back junction cells boosting PV solar cell efficiency in recent years [65]. Crystalline polysilicon is the current dominant technology for PV modules, with a more than 95% market share. The use of efficient cell design (PERC) is also increasingly dominant with an almost 75% market share [66]. New technologies are under development, such as perovskite solar cells, dye solar cells, and even quantum dot solar cells, among others [67] with the expectation of promising efficiency results in the future.

This work discusses the research we have conducted on UV degradation and conversion efficiency, and photoluminescence studies of lanthanide ions for use in solar cells as spectral down-shifters to tackle these challenges. The rest of this work is structured as followed: First, the materials and methods used to synthesize and characterize the studied complexes are given. Next, the obtained results are detailed, including structural studies, the preparation of down-shifting films, absorption spectra, and photoluminescence results. Finally, these DS films are applied in different photovoltaic devices, and the external quantum efficiency is calculated for each configuration. We also include studies conducted on the UV degradation of these DS films and their use in bifacial photovoltaic cells.

## 2. Materials and Methods

### 2.1. Materials

**Figure 2:**
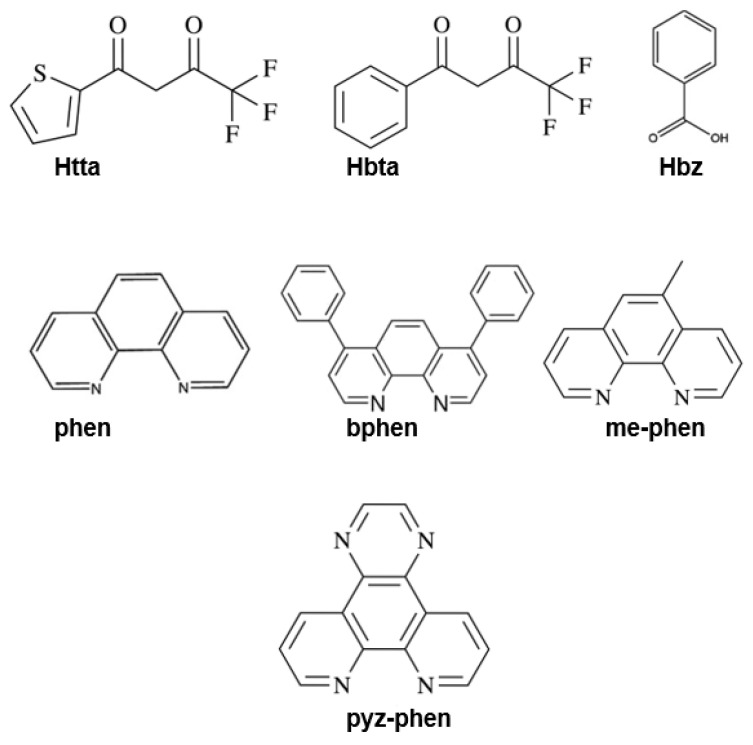
Schematic structures of thenoyltrifluoroacetone (Htta), 1-benzoyl-3,3,3-trifluoroacetone (Hbta), benzoic acid (Hbz), phenanthroline (phen), 4,7-biphenyl-1,10-phenanthroline (bphen), 5-methyl-1,10-phenanthroline (me-phen) and pyrazino [2,3-f][1,10]phenanthroline (pyz-phen) ligands.

Unless otherwise stated, substances purchased from commercial sources were used without further purification. All the synthetic procedures were performed under a dinitrogen atmosphere to avoid oxidation of the reagents. The chemicals and reagents used were ethanol, acetonitrile, n-hexane, n-pentane, dichloromethane, and triethylamine (98%). The ligands used as antennas were fluorinated β-diketonates, such as (Hbta, 99%) and (Htta, 99%), benzoic acid (Hbz, 99.99%), and the derivatives of phenanthroline (phen, 99%), such as (bphen, 97%), (me-phen, 99%), and (pyz-phen, 99%). The structures of these ligands are shown in Figure 2.

The Ln(III) nitrates were Eu(NO_3_)_3_·5H_2_O (99.99%), Gd(NO_3_)_3_·6H_2_O (99.99%), Tb(NO_3_)_3_·5H_2_0 (99.99%), Yb(NO_3_)_3_·5H_2_0 (99.99%), and the corresponding oxide Eu_2_O_3_ (99.99%). PMMA, average Mw 996,000, and UV-transparent EVA were used as an oxygen-impermeable matrix for encapsulating the optically active dye. Commercial glass substrates consisting of thin, flat pieces of glass (20 × 20 mm and 1 mm thick from Pilkington, and 1 × 1 inch and 1 mm thick from Technical Glass Products) were used for these complexes.

### 2.2. General Methods

Elemental analyses (of C, H, N, S) and Fourier Transform Infrared (FT-IR) spectra (as KBr disks, between 400 cm^−1^ and 4000 cm^−1^) were acquired using a FLASH EA 1112 CHNS-O microanalyzer and a Thermo NicolletAvatar 360 FT-IR spectrometer (Thermo Fisher Scientific, Waltham, MA, USA), respectively.

UV-visible spectra between 220 nm and 800 nm were recorded on a Varian Cary 50 bio UV-visible Spectrometer with samples dissolved in ethanol.

X-ray powder diffraction patterns were recorded on a PANanalytical X’pert X-ray diffractometer with 1.54184 Å Cu-Kα radiation, at room temperature. Single-crystal X-ray diffraction data were collected with an Agilent SuperNova diffractometer with a micro-focus X-ray at the same radiation wavelength. The structure was solved by direct methods with SHELXS-2016 [68].

### 2.3. Photoluminescence and EQE Measurements

For the PL measurements, emission spectra were acquired using an AvaSpec-2048 spectrometer, a 300 W Xe arc lamp passed through a 0.25 m double-grating monochromator detecting with a 0.25 m double-grating monochromator with an R928 photomultiplier, and an FLS 1000 spectrometer. The lifetimes were measured with a LeCroy WS 424 Oscilloscope using an EKSPLA/NT342/3/UVE Optical Parametric Oscillator (OPO) laser to excite the sample. The absolute fluorescence quantum yields (PLQY) were calculated using the ‘Indirect Method’ with an FLS1000 fluorimeter (Edinburgh Instruments Ltd, Livingston, UK). For the EQE measurements, a SPECLAB commercial setup was used, and UV-to-IR optical radiation was recorded with the Oriel Merlin Digital Lock-in radiometry system (control unit, preamplifier, and optical chopper 8–1100 Hz) with a sensitivity of 0.5 μV. The typical values measured are in the range of 10 mV. A dual Xenon and Quartz Halogen Source (250–2500 nm) housing a 75 W short-arc xenon lamp and a 100 W quartz halogen lamp with automated selection via an Oriel Cornerstone 260 motorized high-resolution 1/4 m monochromator scanning from 280 nm to 2200 nm was used for the measurements.

### 2.4. Accelerated Aging

A XENOCLIMA-1500RF CCI P/2999 chamber was utilized at ULL to assess the UV degradation of the PV module’s DS layers. The chamber incorporates two optical glasses that permit the transmission of UVA radiation (300–400 nm), which is known to cause the most significant photochemical aging. The chamber’s cooling system effectively limits the conversion of IR radiation into heat. Within the climatic chamber, the spectral irradiance in the 350–400 nm range measures 227.47 W/m^2^, which is approximately 2.4 times higher than the AM1.5G solar irradiance in the same range [69]. To prevent any potential cross-effects during the experiments, the temperature and relative humidity were maintained at 22 °C and 30%, respectively.

## 3. Results and Discussion

### 3.1. Synthesis and Characterization of the Complexes

The synthesis of all the complexes, except for C4, has been previously described in our prior works [24,25,26,27,28,29,30,31,32,33]. For the purpose of discussing the general synthesis strategy of these complexes, we will focus on the synthesis of the new complex C4. The complex [Eu(bta)_3_pyz-phen] (C4) is obtained by the reaction of stoichiometric quantities of Eu(NO_3_)_3_·5H_2_O, pyz-phen, Hbta, and triethylamine (C_2_H_5_)_3_N), (99%). 1-benzoyl-3,3,3-trifluoroacetone (C_10_H_7_F_3_O_2_) (648 mg, 3 mmol) was dissolved in 40 mL of ethanol, and the solution was heated at 65 °C under stirring. Next, (C_2_H_5_)_3_N) (416 μL, 3 mmol) was added under stirring. Then, a solution of pyz-phen (208 mg, 1 mmol) in ethanol (4.0 mL) was added. Meanwhile, in a separate container, Eu(NO_3_)_3_·5H_2_O(425 mg, 1 mmol) was dissolved in ethanol (10 mL). Finally, both solutions were mixed and stirred for 3 h. After that time, 50 mL of water was added, and a white product was obtained, which was filtered, washed with water, and dried in an oven at 60 °C overnight (yield, 92%).

The complexes were prepared under a dinitrogen atmosphere in order to avoid oxidation of the reagents. All complexes were prepared from stoichiometric amounts of their components. Initially, the syntheses were carried out in water, but we subsequently found that we obtain higher yields when the synthesis is performed in ethanol. Ligands with diketonate groups need to be deprotonated to obtain the corresponding diketonate, and the best result is achieved using triethylamine as a base. The optimal reaction time is 180 min. The crude product can be obtained by the addition of water, but it is highly recommended to recrystallize it by liquid–vapor diffusion. Chloroform, THF, or acetonitrile can be used as solvents, and n-hexane or n-heptane as precipitating agents. By using these procedures, single crystals and powder can be obtained, which are characterized by X-ray diffraction techniques. The purified products are used for photoluminescence measurements and film preparation.

### 3.2. Structure of the Complexes

We have included diffraction patterns of the different complexes in the Supplementary Material (Figures S1–S7). The structures of complexes C1 and C2 consist of centrosymmetric dinuclear molecules in which each Eu atom is bound to one phenanthroline or bephenanthroline molecule and one terminal bidentate benzoate ligand (Table 1). The powder diffraction pattern and selected atomic distances of complexes C1 and C2 can be found in previous works [24,25]. These complexes crystallize in the triclinic system, space group *P1*. Data on the structure of complex C3 in single-crystal form have not been published, and we have not measured its structure. However, we have found that the complex with ligand 1,10-phenanthroline (phen), namely Eu(tta)_3_phen, belongs to the triclinic crystal system with space group *P1* [70].

The asymmetric unit of complex C4 contains two different molecules of complex C5, which are stereoisomers. The two molecules have the same organic ligands, the same coordination number, and the same coordination environment of the central Eu(III) ion. However, the two molecules differ in the relative orientation of one of the bta- ligands. Complexes C4 and C5 crystallize in the orthorhombic *Pna21* and *Pbca* space groups, respectively. Complex C6 crystallizes in the monoclinic *P21/c* space group with its Eu(III) ion bound to one 5-methyl-phenanthroline and three β-diketonate benzoyl trifluoroacetone ligands.

The structures of complexes C7–11 are shown in Table 2. The crystal structure of C8 was solved by single-crystal X-ray diffraction and it was found to be isostructural with the corresponding complexes C7 and C9. Complex C8 crystallizes in the monoclinic P21/c space group and has a molecular structure in which the Yb(III) ion is bound to six oxygen atoms of three β-diketonate bta- ligands and two nitrogen atoms of one 5-methyl phenanthroline ligand. C10 crystallizes in the orthorhombic *Pna21* space group and has a molecular structure with the Yb atom surrounded by three bta- β-diketonate ligands and one 2,3-pyrazine-1,10-phenanthroline ligand. C11 crystallizes in the orthorhombic *Pbca* space group, and its molecular structure consists of Er(III) ions surrounded by three tta- β-diketonate ligands and one pyz-phen ligand.

In the case of the [M1M2(bz)_4_(tta)_2_(phen)_2_] series, PXRD patterns confirmed that all the complexes were isostructural. The structure of C12 (Table 3), solved by single-crystal X-ray diffraction, showed that these complexes crystallize in the triclinic $P\bar{1}$ space group as neutral centrosymmetric dinuclear molecules of [M1M2(bz)_4_(tta)_2_(phen)_2_] with four bridging benzoate (bz−), two 2-thenoyltrifluoroacetonate (tta−), and two 1,10-phenanthroline (phen) ligands.

### 3.3. Preparation of the Luminescent Down-Shifting Layers

Samples containing 1–7 mg of complexes C1–2 were dissolved in 7 mL of CH_2_Cl_2_ in a refrigerated ultrasonic bath, then 70 mg of PMMA was added and the mixture was stirred until the polymer was completely dissolved, obtaining samples with relative concentrations of the actives species ranging from 1.4 to 10%. The spin-coating method was then used for film deposition (50–100 μm) on commercial glass substrates.

An analysis of the additional costs of a solar panel with this type of film is given in previous studies [26], where we estimated the per square meter (m^2^) cost a of solar panel with 15% efficiency to be approximately 0.05 EUR/m^2^ for every 1% of complex product used. However, these costs were calculated in 2015, and the prices of the chemicals used have increased significantly in recent years. Thus, for complex C1, we have made a revised estimate of its price per square meter for the same concentration of approximately 0.48 EUR/m^2^. For the other complexes, we have also estimated their price in euros per square meter for a 1% concentration. From lowest to highest price, the C1–6 series costs consist of as follows: C1 = 0.48; C6 = 0.84; C5 = 1.24; C4 = 1.28; C3 = 1.71; C2 = 2.28. For the C7–11 series, C7 = 0.56; C8 = 0.58; C9 = 0.61; C11 = 0.97; C10 = 1.02. For the C12–17 series, the equivalent figures are: C13 = 0.16; C15 = 0.32; C16 = 0.36; C14 = 0.37; C17 = 0.42; C12 = 0.49.

To obtain the DS layers corresponding to complexes C3 and C7, and the C12–17 series before encapsulating them on a PV mini-module, Deltalab 1.1 mm thick EUROTUBE^®^ slides (25 mm × 70 mm) were cut into 20 × 20 mm squares or 25 × 20 mm pieces and immersed in a 2 M sulfuric acid solution for one hour, rinsed with a generous amount of deionized water, and dried at 60 °C overnight. A total of 10 mg of DS complex mixed with 25 mg of EVA was dissolved in 1.5 mL of CH_2_Cl_2_ under vigorous stirring for 20 min. Then 300 µL of that solution was dropped onto the glasses and the solvent was evaporated at room temperature.

**Figure 3:**
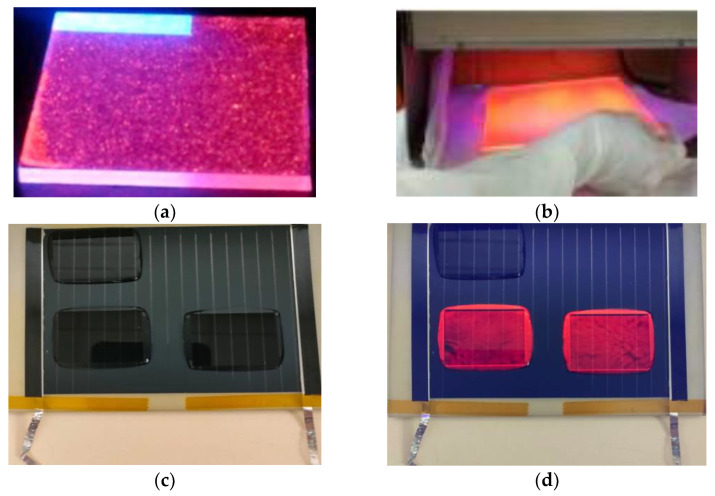

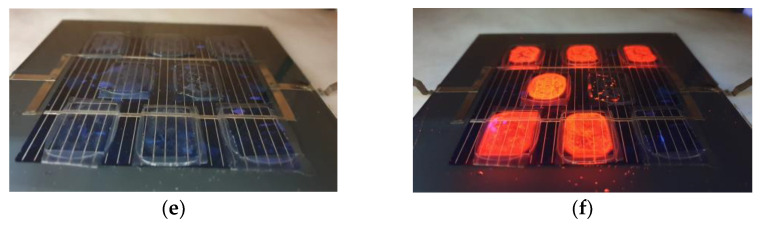
Images of down-shifter layers corresponding to complexes C1 (**a**) and C3 (**b**) when irradiated with UV light. Photograph (**c**) shows down-shifter layers of complex C7 laminated with EVA on a mini-module of copper indium gallium selenide (CIGS), while photograph (**d**) shows these layers when illuminated with UV light. Photograph (**e**) shows converter layers from the C12–17 series laminated with EVA on a multi-crystalline Silicon mini-module, while photograph (**f**) shows the same samples when irradiated with UV light.

Figure 3a,b display the down-converting layers of complexes C1 and C3, respectively, which are embedded in PMMA and deposited on bare glass substrates under UV radiation. The C3–7 and C12–17 series samples were encapsulated using a standard procedure [71] using a Lab PV Module Laminator from Fraunhofer ISE. Figure 3a,b show samples of complex C1 and C3, respectively, exposed to UV light. Figure 3c,d show examples of the down-converting layers of complex C7 embedded in EVA, without and with UV light. Figure 3e,f show examples of the down-converting layers of complexes C12–17 embedded in EVA, without and with UV light.

### 3.4. UV-Vis Absorption Spectra

By using the values for the absorbance A obtained, using the UV-visible spectrometer specified in the general methods, along with the concentration of the absorbing species per unit volume c [M = mol/L] and the distance that the light travels through the solution l [cm], we can determine the molar absorptivity *ε* = (A/lc) [M^−1^ cm^−1^].

**Figure 4:**
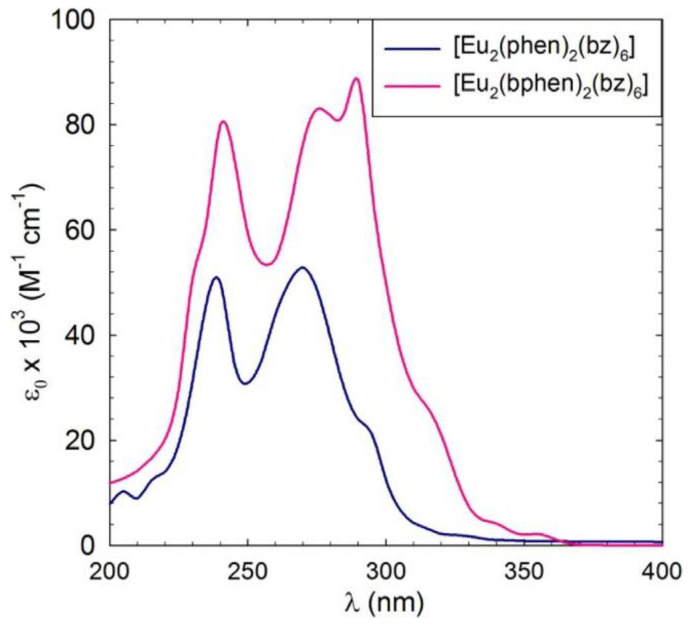
UV-vis spectra of complexes C1 and C2: [Eu_2_(phen)_2_(bz)_6_] and [Eu_2_(bphen)_2_(bz)_6_] in CH_2_Cl_2_ at c ≈ 2.5 × 10^−6^ M.

The UV-vis absorption spectra of complexes C1 and C2 are presented in Figure 4 and show that, compared to C1, C2 shows an increase in the molar absorption coefficient ε, with a maximum at 290 nm. This increase is usually correlated with an increase in the emission since the bphen ligand itself exhibits higher absorption compared to phen.

**Figure 5:**
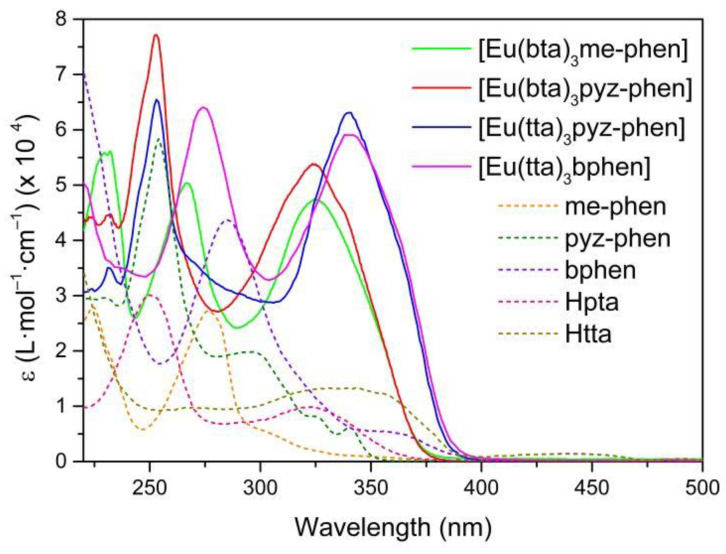
UV-vis absorption spectra of the Hbta, Htta, bphen, me-phen, and pyz-phen ligands together with [Eu(tta)_3_bphen] (C3), [Eu(bta)_3_pyz-phen] (C4), [Eu(tta)_3_pyz-phen] (C5), and [Eu(bta)_3_me-phen] (C6) complexes in ethanol at c ≈ 10^−5^ M.

The UV-vis absorption spectra of complexes C3–6 are shown in Figure 5. They are considerably enhanced compared to the ligand spectra since the complexes contain three molecules of the diketonate ligands plus the phenanthroline derivative. The pattern of the absorption of [Eu(bta)_3_pyz-phen] and [Eu(bta)_3_me-phen] complexes are very similar around 320 nm since all the complexes contain the same diketonate ligand and the contribution of the phenanthroline derivative is expressed at lower wavelengths. [Eu(tta)_3_bphen] and [Eu(tta)_3_pyz-phen] have a higher absorption coefficient at higher wavelengths due to the more intense absorption of the tta- ligands at higher wavelengths (340 nm). All the complexes have little absorption in the visible region, and diluted solutions are almost colorless.

**Figure 6:**
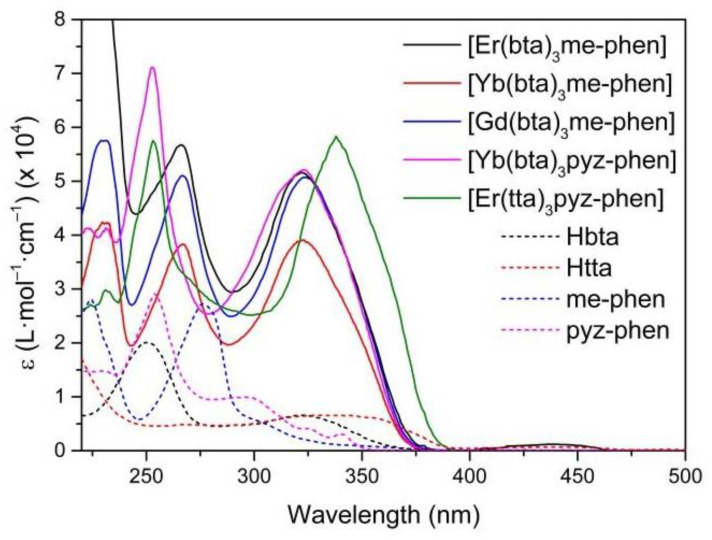
UV-vis absorption spectra of the Hbta, Htta, me-phen, and pyz-phen ligands together with Yb(III) and Er(III) complexes in ethanol at c ≈ 10^−5^ M.

Figure 6 shows the ε vs. λ spectra of complexes C7–11 together with the free ligands Hbta, Htta, me-phen, and pyz-phen in the UV-vis region.

Complexes C7–10 have the same band at 320 nm due to the bta- ligands, while complex C11 presents a band at 340 nm corresponding to the absorption of the tta- ligands. Since these absorbances can be attributed to the S_0_ → S_1_ transition of the ligands, they give the energies of their singlet excited states, being in this case 26,700 cm^−1^ and 23,500 cm^−1^ for the bta- and tta- ligands, respectively, in agreement with previously reported values [72]. The contribution of the phen-derivative ligands appears at lower wavelengths. Complexes C10 and C11 exhibit a maximum at around 250 nm due to the pyz-phen ligand and complexes C1–3 have two bands at around 230 nm and 270 nm corresponding to the maxima of the me-phen ligand.

**Figure 7:**
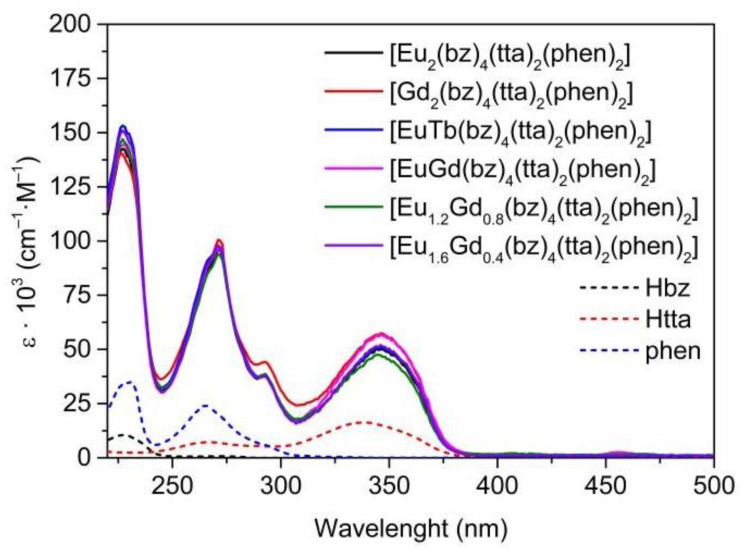
UV-vis absorption spectra of the Hbz, Htta, and phen ligands together with [M1M2(bz)_4_(tta)_2_(phen)_2_] complexes in ethanol at c ≈ 10^−5^ M.

In the case of the [M1M2(bz)_4_(tta)_2_(phen)_2_] complexes, all present the same pattern since they are analogous complexes with bz-, tta-, and phen ligands (Figure 7). The increase in the complexes’ absorption compared to the free ligands is greater than in the previous complexes since each complex molecule contains two molecules of phen, four bz- molecules, and two β-diketonate tta- ligands. The first absorption band at 227 nm with a shoulder at 230 nm is due to the contribution of the bz- and phen ligands, respectively. Phenanthroline molecules cause the second maximum at 271 nm with a shoulder at 292 nm, and the last band at 346 nm experiences a bathochromic shift from the absorptions of the tta- ligands at 336 nm.

### 3.5. Photoluminescence

Ternary complexes with β-diketonate ligands are a good choice of organic antennas to achieve efficient energy transfer from T1 to Ln^3+^ ion, as many previous studies indicate [73]. The derivatives of phenanthroline are also very efficient for their rigidity and the direct bond of the nitrogen atoms to the metal ion when the complex is formed. The photoluminescence quantum yield, also known as PLQY or Φ [19], of a molecule or material, is defined as the ratio of the number of photons emitted to the number of photons absorbed (internal PLQY). It is also important to know the fluorescence lifetime (τ) of a molecule, which is defined as the average length of time it spends in the excited state. These properties of a fluorophore or fluorescent molecule are crucial for understanding the behavior and interactions of many important materials at the molecular level. The dependence of the quantum yield on the influence of a ligand-to-metal charge-transfer state is determined by its energy level relative to both the ligand and metal-ion levels, as well as the significance of the cross-over between this state and the excited and ground term manifolds of the Ln(III) ion [3].

Studying the UV-vis absorption and photoluminescence properties gives us the necessary data to estimate the energy diagrams of the luminescence processes. This includes the energy of the excited states, the intersystem crossing (ISC), and the energy transfers (ET) of the down-shifting processes, among others. We have included the energy diagrams of the different compounds in the Supplementary Material (Figures S8–S11).

**Figure 8:**
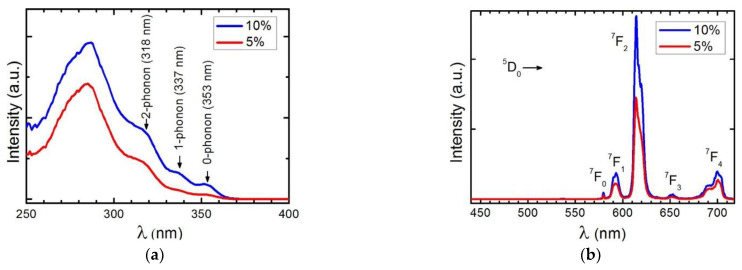
(**a**) Antenna excitation between 250 and 400 nm and (**b**) corresponding Eu^3+^ emission spectra of complex C2 at 5% (red line) and 10% (blue line) in PMMA.

The photophysical measurements were performed with the samples as solids in order to avoid solvent effects. Photoluminescence excitation (PLE) and emission (PL) spectra of C1 in PMMA at 2.0 weight% (wt%) placed on glass are reported in our previous work [26], while the corresponding spectra of C2 at 5.0 wt% and 10 wt% in PMMA films are shown in Figure 8 [27].

**Figure 9:**
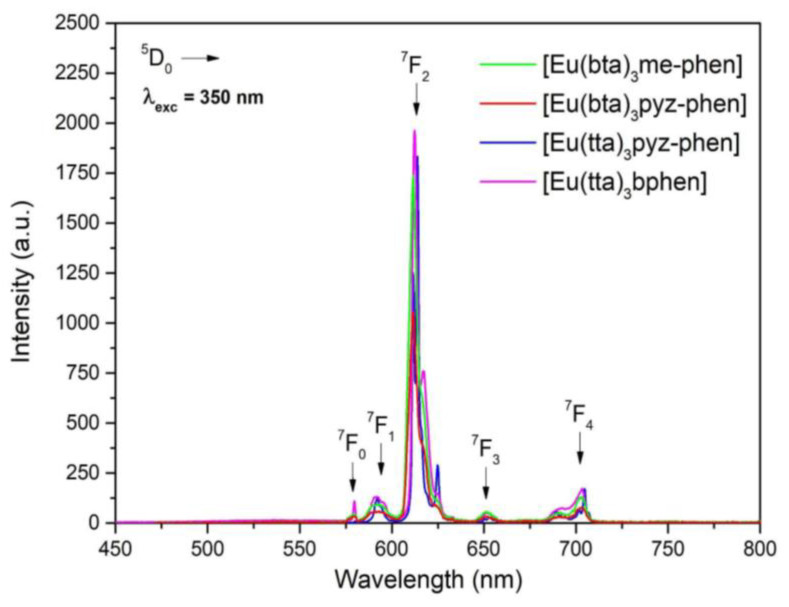
Emission spectra of complexes C3–6 obtained under excitation with a xenon lamp at 350 nm.

The excitation spectra of C2 display a broad band between 250 and 350 nm with a maximum at 280 nm. The emission spectra reveal the characteristic emission peaks of the Eu3+ ion corresponding to the ^5^D_0_→^7^F_0-4_ transition, with the emission at 614 nm being the most intense transition. Analogous to these PL spectra, the emission spectra excited at 375 nm of complexes C3-5 (Figure 9) show five sharp lines at 579, 590, 612, 652, and 701 nm, due to the ^5^D_0_→^7^F_0-4_ transitions of Eu^3+^. Of these transitions, the most intense is ^5^D_0_→^7^F_2_ at 612 nm, which is responsible for the red emission color.

**Figure 10:**
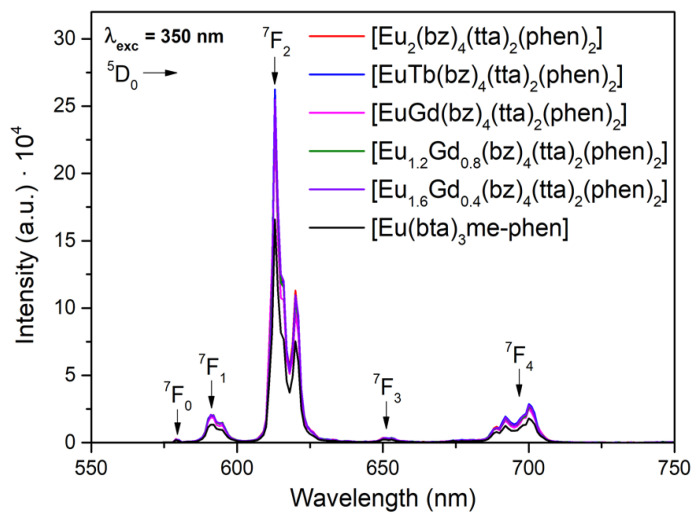
Emission spectra of [Eu(bta)_3_me-phen] and all [M1M2(bz)_4_(tta)_2_(phen)_2_] containing Eu(III) ion obtained under excitation with a xenon lamp at 350 nm.

All the complexes C6, C12, and C14–17, which contain the Eu(III) lanthanide ion, exhibit intense emission with sharp peaks in the 570–730 nm range associated with the ^5^D_0_ → ^7^F_0–4_ transitions of the Eu(III) ion (Figure 10).

**Figure 11:**
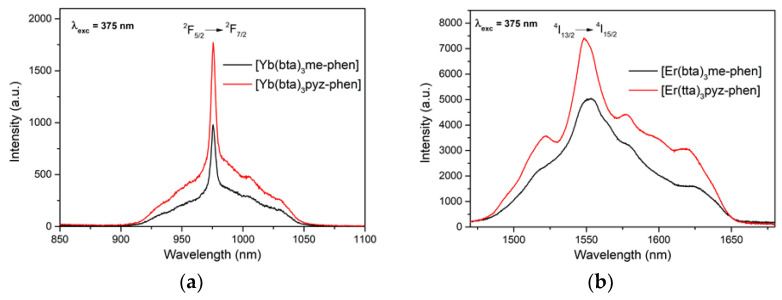
Emission spectra of (**a**) [Yb(bta)_3_me-phen] and [Yb(bta)_3_pyz-phen] and (**b**) [Er(bta)_3_me-phen] and [Er(tta)_3_pyz-phen] exciting with a 375 nm laser.

The emission patterns of complexes C8 and C10, containing [Yb(bta)_3_me-phen] and [Yb(bta)_3_pyz-phen], respectively, were measured with excitation at 375 nm, and show an intense NIR emission centered at 980 nm that corresponds to the typical ^2^F_5/2_ → ^2^F_7/2_ transition for Yb(III) (Figure 11a). The compounds [Er(bta)_3_me-phen] and [Er(tta)_3_pyz-phen] also have their principal emission band in the NIR region, with a maximum at 1550 nm due to the ^4^I_13/2_ → ^4^I_15/2_ transition of the Er(III), when excited at 375 nm (Figure 11b).

One of the crucial design considerations for thermally activated delayed fluorescence (TADF) molecules is achieving a narrow energy gap between the S_1_ and T_1_ states (∆ε_ST_). TADF has garnered significant attention due to its ability to enhance the efficiency of organic light-emitting diodes (OLEDs) by effectively utilizing triplet excitons [74]. In the Supplementary Material (Table S1), Table S1, we include the energies of the first singlet (ES_1_) and triplet (ET_1_) excited states relative to their corresponding highest occupied molecular orbital (HOMO) for the ligands used, along with the bibliographic reference to our previous works and external sources.

**Figure 12:**
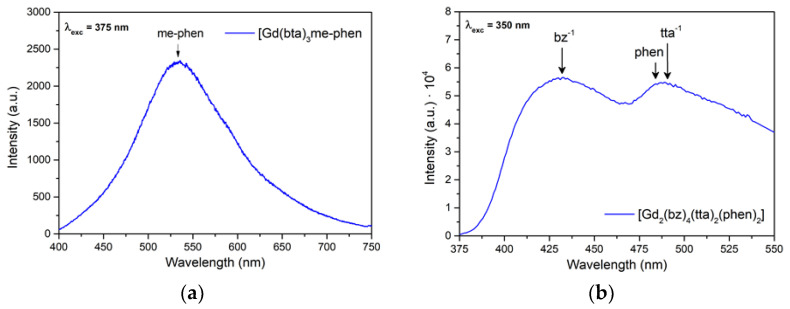
Emission spectra of (**a**) [Gd(bta)_3_me-phen] excited with a 375 nm laser and (**b**) [Gd_2(_bz)_4_(tta)_2_(phen)_2_] excited with a xenon lamp at 350 nm.

Figure 12 shows the emission patterns of complexes C9 and C13, [Gd(bta)_3_me-phen] (a) and [Gd_2_(bz)_4_(tta)_2_(phen)_2_] (b), where the phosphorescence of me-phen, bz^-^, tta^-^, and phen is observed. Normally, the energy of the excited levels of Gd(III) is above the energy of the excitation radiation or the energies of the excited states of the ligands. In this case, the excitation wavelength was 350 nm, that is 28,570 cm^−1^, while the energy of the first excited state of Gd(III) (^6^P_7/2_) is around 32,500 cm^−1^. In this case, the incident power from the laser is sufficiently strong to observe phosphorescence even at room temperature. We compared the emission spectrum bands of the Gd(III) complexes with the triplet values of the corresponding ligands found in the literature and confirmed their consistency [72,75,76,77].

In Table 4, we summarize the measured values of lifetime represented by decay intensity [32,78] and values of PLQY using indirect methods and an integrating sphere [33] for some compounds. In this approach, the quantum yield is calculated using Equation (1)

(1) $$PLQY=\frac{S_{A}\left(E_{c}-E_{A}\right)-S_{C}(E_{B}-E_{A})}{S_{A}(S_{B}-S_{C})}$$

where *S* and *E* represent the integrals of the excitation peak and emission of the sample, respectively. The subscripts *A*, *B*, and *C* indicate the measurements for the direct excitation of the blank, direct excitation of the sample, and indirect excitation of the sample, respectively. In some cases, although we have not yet measured these values, we did address their studies as down-shifting layers in photovoltaic mini-modules, since the characterization of its photoluminescence had already demonstrated the suitability of the complex for PV applications.

### 3.6. EQE Measurements

To quantify the effect of the DS layers on top of the PV devices, we calculated the increase in photogenerated current, Δ*J_sc_*, as a function of the wavelength, *λ*, through Equation (2) [81]

(2) $$\Delta J_{sc}=q\int \phi (\lambda )\cdot \Delta EQE(\lambda )d\lambda =\int E_{0}\cdot \Delta SR(\lambda )d\lambda$$

where *q* represents the charge of an electron, *ϕ* is the incident photon flux, Δ*SR* is the increase in spectral response (SR), and *E*_0_ is a standard reference of solar spectral irradiance based on ASTM G173-03 [69]. The spectral response uses the power of the light at each wavelength, with its magnitude given in Ampere/Watt. Converting *EQE* to *SR* is done using the formula *SR* = *EQE*·λ (nm)/1239.8. Equation (2) was used to determine the current density *J_sc_* of the solar cell with DS films encapsulated. Estimations of the relative increase in conversion efficiency (*η*) are made while considering that the open circuit voltage (*V_oc_*) and the fill factor (*FF*) remain constant when the down-shifter is placed on the PV mini-module. By assuming that (%) is equal to the product of *J_sc_*, *V_oc_*, and *FF*, for one sun of irradiation, the increment in relative percentage of conversion efficiency, (Δη/η) %, can be calculated using the following expression

(3) $$(\Delta \eta /\eta )\times 100\;≳\;(\Delta J_{sc}/J_{sc})\times 100$$

Below, we summarize the results we have obtained with the complexes used for different experimental configurations. In order to optimize our research on the use of DS films in different PV devices, we have been testing the placement of photoluminescent films on the device, the concentration of materials used in their manufacturing, the light concentration using spherical reflectors, the encapsulation of the photoluminescent films on the device, and the degradation they may undergo with prolonged exposure to ultraviolet light.

#### 3.6.1. DS Layers Using Different Concentrations of Complexes C1 and C2

**Figure 13:**
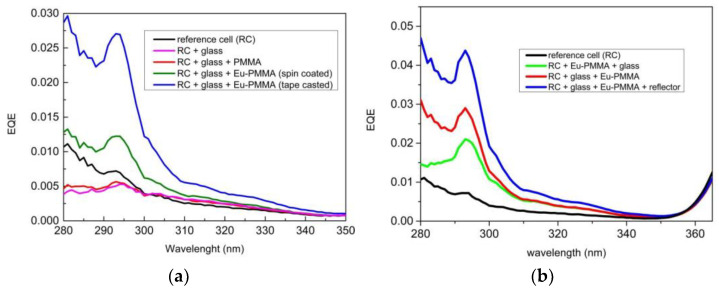
EQE values ranging between 0 and 1 were obtained for the 280–350 nm spectral range for various experimental configurations of the DS layer of complex C1 placed on the reference cell (RC). (**a**) The EQE behavior was analyzed when the DS layer was deposited through spin-coating or tape-casting on glass and (**b**) Additionally, the EQE behavior was studied when the DS layer was deposited through tape-casting on the rear side of the glass and covered by a UV reflector.

Figure 13 shows the measurements obtained in EQE when using a DS film of complex C1 for different experimental configurations. Figure 13a shows the EQE behavior for the 280–350 nm spectral range when the DS layer was deposited through spin-coating or tape-casting on glass while Figure 13b shows the wavelength dependence of the EQE when the DS layer was deposited through tape-casting on the rear side of the glass and covered by a UV reflector.

The analysis of the measurements obtained of the EQE with the DS film of complex C1 allows us to calculate estimates of the relative increase in percentage efficiency, as shown in Table 5.

The values of (Δ*η*/*η*) % are very high in the UV region where the photovoltaic device practically has no response. This increase is what can ultimately lead to a gain across the entire studied spectrum.

**Figure 14:**
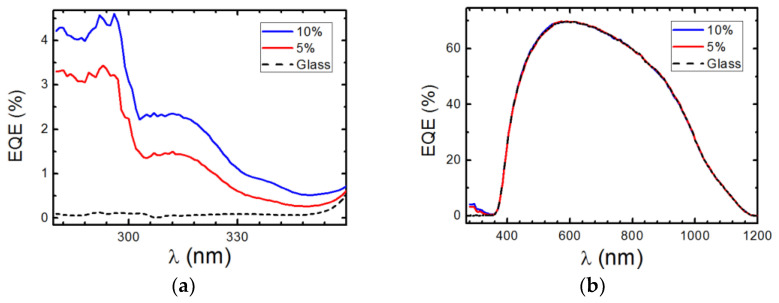
(**a**) EQE values in percent measured for the 280–350 nm spectral range and (**b**) for the 280–1200 nm spectral range when the reference solar cell (RSC) is covered with the clean glass (black dashed line) and with 5% (red line) and 10% (blue line) of complex C3.

Complex C2 using the bphen ligand shows improved absorption and photoluminescence results compared to complex C, as shown by the results discussed earlier. The measurement of the EQE of the device with DS films continued with higher concentrations of complex C2, as shown in Figure 14a,b. Figure 14a displays the obtained EQE measurements for the UV region, where there is a maximum of 4.6% and 3.5% for complex C2 concentrations of 10% and 5%, respectively. The EQE measurements across the spectrum shown in Figure 14b demonstrate that these DS films do not show reduced efficiency in the visible region of the spectrum.

#### 3.6.2. DS Layers Using Different Concentrations of Complex C3

**Figure 15:**
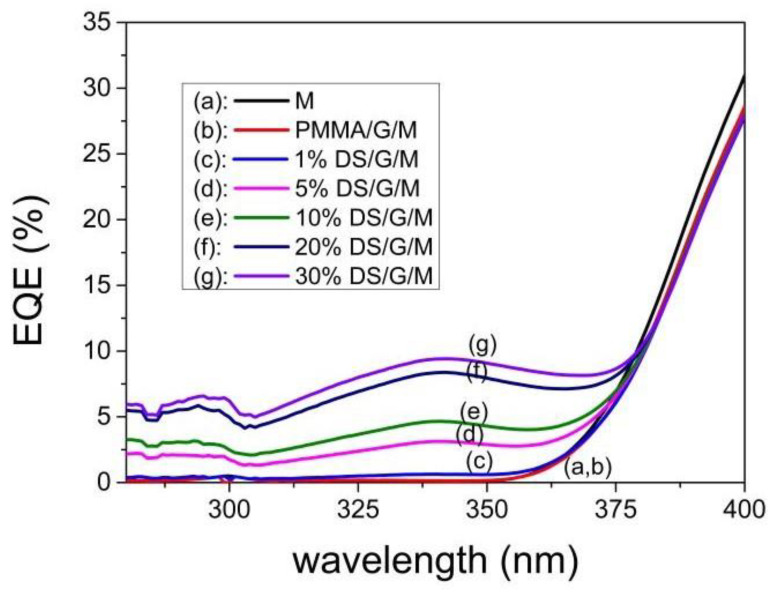
EQE values in percent for the 280–400 nm spectral range were measured for different concentrations (ranging from 1% to 30%) of complex C3 embedded in a thin PMMA film of the DS layer. The film was deposited on a glass substrate (G) and placed on the PV mini-module (M).

When the PV mini-module used has virtually no response in the UV region, the EQE values increase with the concentration of complex C3 when the DS film is placed on top. This evolution can be studied based on the results shown in Figure 15. Thus, for the maximum EQE value at an excitation wavelength of 340 nm, it is found that there is a maximum concentration value of complex C3 in the DS film of close to 30%, as above that value the transmittance of the DS film decreases to zero.

**Figure 16:**
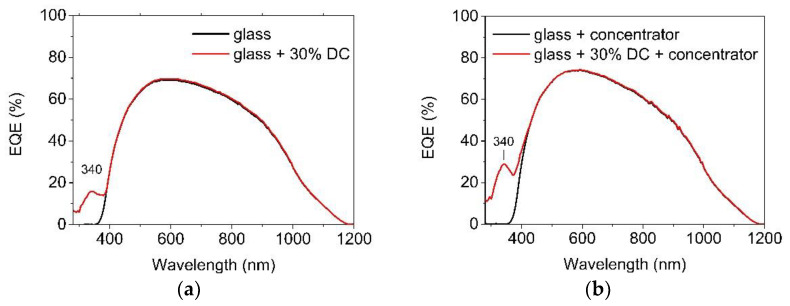
(**a**) EQE values, expressed as a percentage, measured for the spectral range of 280–1200 nm using a 30% concentration of complex C3 in the down-converter (DC) layer following the experimental configuration for the values obtained in Figure 14. (**b**) The EQE values measured when the DC layer was covered by a hemispherical reflector (concentrator).

The maximum down-shifting efficiency is determined by increasing the concentration of the active species to its solubility limit [27]. Our results are consistent with the EQE behavior, as shown in Figure 16.

The increments in the relative percentage of conversion efficiency from Figure 16a were 1.07% and 1.92% for 10% and 30% concentrations of complex C3, respectively. For the PV mini-module based on a single p-type mc-Si solar cell with a 16% conversion efficiency, the increases were 0.17% and 0.31%, respectively.

If we use the same configuration but add a hemispherical reflector (concentrator) on top of the down-shifter to collect most of the down-shifted photons emitted isotropically out of the device, we obtain the EQE values shown in Figure 16b. With a 30% concentration of complex C3, the relative percentage of conversion efficiency increased by 2.88% and the absolute conversion efficiency increased by 0.46%. This new configuration can be integrated into PV devices for specific applications related to concentrator photovoltaics.

#### 3.6.3. DS Layers Using Complexes C4–6

**Figure 17:**
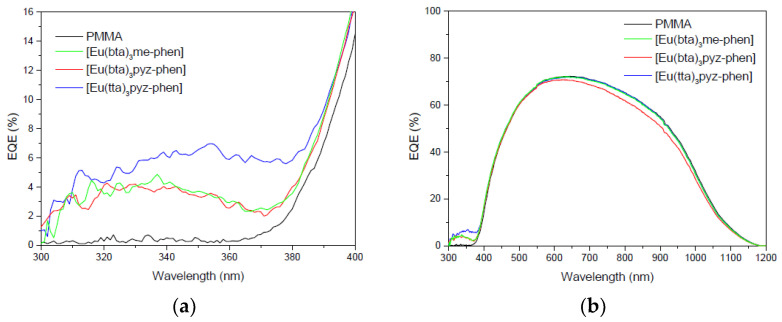
(**a**) EQE values in percent for the 300–400 nm spectral range and (**b**) for the 300–1200 nm spectral range were measured for the DS layers of complexes C4–6.

Luminescent down-shifting (LDS) layers containing the complexes C4, C5, and C6 embedded in polymethylmethacrylate (PMMA) have been placed on top of the mini-module to study their photovoltaic conversions compared to the PMMA when there is not complex. Figure 17a shows a positive increase in EQE values for the 300–400 nm spectral region and Figure 17b shows the EQE values for the 300–1200 nm spectral region.

The EQE measurements shown in Figure 17b show that the use of complexes C5 and C6 as DS layers (blue and green lines) can improve the efficiency as we will show in the next sections.

#### 3.6.4. DS Layers Encapsulated Using Complexes C6 and C12–17

First, the DS films were just put on top of the PV devices without any encapsulation process, resulting in an increase in the EQE for the 310–370 nm spectral range. However, in the region of the solar spectrum where the mini-modules have a good response (380–1100 nm), the DS films show reduced efficiency due to poor transmittance, which indicates that the encapsulation of the films on top of the PV devices was necessary.

**Figure 18:**
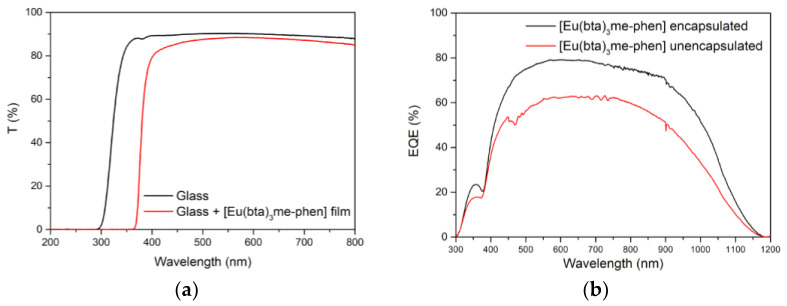
(**a**) Transmittance (T) values in percent for the UV-vis spectral range of the bare glass (black line) and complex C6 embedded in EVA and deposited on the glass (red line) and (**b**) EQE values in percent for the 300–1200 nm spectral range measured for the DS layer of complex C6 before and after encapsulating.

Initially, the films were made with PMMA as mentioned above, but it wasn’t possible to obtain clear and homogenous luminescent DS films after the encapsulation process using this approach. EVA is the main encapsulant polymer used in the fabrication process of PV devices, so we tried preparing the films using EVA. Clear DS films that enhanced the EQE of the mini-modules for the 300–370 nm range were obtained using EVA (Figure 18b). These films also eliminated any losses between 380 nm and 1100 nm thanks to a greater transmittance. In addition, encapsulating the films prevents contact between the DS complex and atmospheric dioxygen. This resulted in increased efficiency of the down-shifting process by avoiding non-radiative deactivation of the excited states of the ligands due to interference from the triplet state of the dioxygen molecule. Therefore, the EQE response in the UV region of the spectrum is higher after encapsulation.

**Figure 19:**
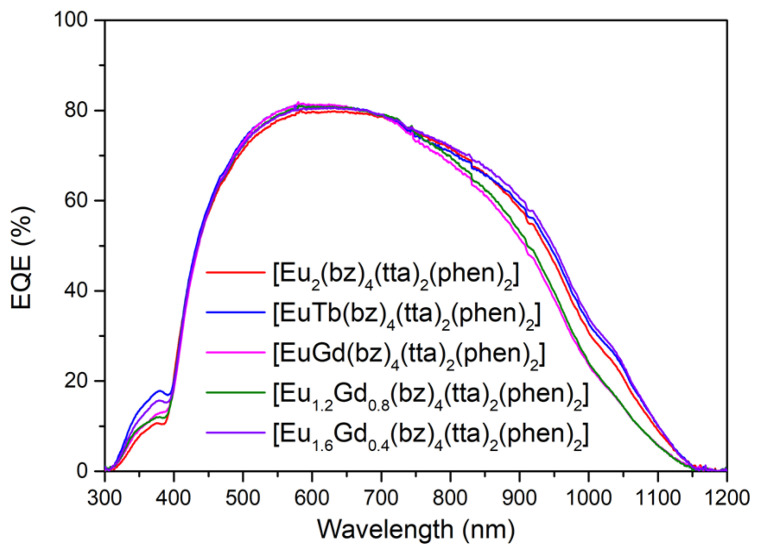
EQE values in percent of the mini-modules with the DS layers of [M1M2(bz)_4_(tta)_2_(phen)_2_] complexes containing Eu(III) ions encapsulated on top.

Once the conditions of the films and the encapsulation process were optimized, the EQE measurements of all complexes encapsulated on top of the mini-module were performed (Figure 19). The greatest increase in EQE was observed in the heterobimetallic complexes C14 and C17, with increases of 16.3% and 14.3%, respectively, for the 300–400 nm spectral range. These complexes were found to exhibit high quantum yields in the photoluminescence study. In the case of complex C6, there was a 10.0% increase in EQE under the same conditions. Complex C6 was subsequently subjected to a UV aging process, as detailed in the next section.

Table 6 shows that all encapsulated LDS layers resulted in a significant increase in both EQE and J*_sc_* within the 300–400 nm spectral range. The highest values were observed for complexes C6, C14, and C17 when they were encapsulated in the mini-module. These three complexes have positively contributed to an increase in the relative percentage of conversion efficiency within the 300–1200 nm spectral range.

In Table 6, we also wanted to show J*_sc_* results through new experiments conducted with intensity–voltage (IV) curve measurements using a solar simulator AM1.5G, using reflectors. An increase in efficiency of the mini-module used for complexes C12–17 was obtained when a hemispherical reflector (concentrator) was placed on top of the down-shifter. We have achieved, in the best-case scenario for the DS layer with complex C14, an increase in conversion efficiency of 0.52% using a PV mini-module of 12.1% efficiency [33].

#### 3.6.5. Accelerate Aging of DS Layers Encapsulated Using Complex C6

**Figure 20:**
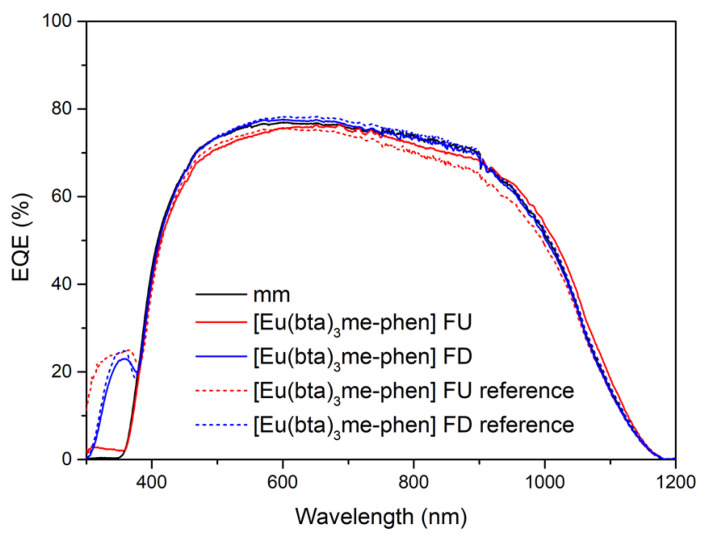
The EQE response of the encapsulated samples evaluated after 2000 h exposure to the xenon lamp within the climatic chamber, covering the spectral range of 300–1200 nm. The dashed lines represent the measurements taken before any UV aging, while the solid lines depict the EQE behavior after exposure to UV radiation.

Cycles of UV radiation were applied to samples of complex C6 for varying durations (2, 5, 10, 50, and 100 h) totaling 2000 h of exposure. Two samples of [Eu(bta)_3_me-phen] encapsulated on top of a mini-module were used, featuring two different film configurations of complex C6 on the glass surface (face-up, FU, and face-down, FD). These samples underwent a study to assess the degradation caused by UV radiation within a climatic chamber. In Figure 20, the EQE curves of the reference samples (non-degraded, without UV exposure) and the study samples after exposure to 450 kW/m^2^ of UV radiation (2000 h in the climatic chamber) are presented. After 2000 h exposure, the FU sample contributes a maximum of only 2.5% to the EQE for the 300–400 nm spectral region. In contrast, the DS-FD sample displayed a greater than 20% contribution at a wavelength of 350 nm compared to the reference sample without the down-shifting complex.

**Figure 21:**
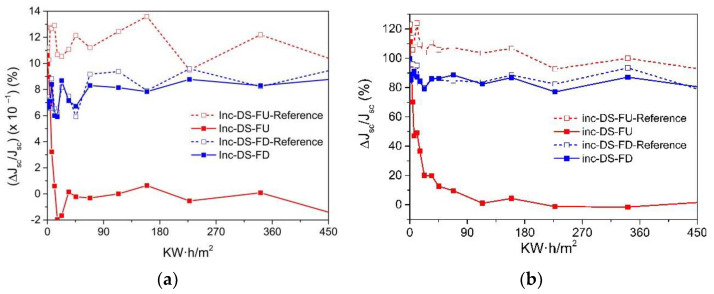
(**a**) The evolution in the relative increase (inc) in the percentage of current density, ΔJ*_sc_*/J*_sc_* (%), after exposure to UV radiation (kW·h/m^2^) (solid lines) compared to samples that have not undergone UV aging (dashed lines), for the 300–120 spectral range and (**b**) for 300–400 nm spectral range.

Figure 21 shows there is a remarkable relative increase in percentage efficiency, (Δ*η*/*η*) (%), when the DS layer encapsulated is not subject to UV degradation, as is the case for the DS-FU sample, of around 1.2% for the 300–1200 spectral range (Figure 12a) and 120% for the 300–400 spectral range (Figure 21b).

After 600 h of UV radiation exposure, the DS-FU sample shows no significant relative increase in efficiency. Conversely, the DS-FD sample demonstrates minimal UV degradation, with a relative increase in efficiency (Δ*η*/*η*) exceeding 80% for the 300–400 nm spectral range and approximately 0.8% for the 300–1200 nm spectral range.

#### 3.6.6. Application of Complex C6 to a Bifacial Solar Cell

In this section, we report initial studies conducted with bifacial mini-modules using one of the most studied complexes from the previous sections, complex C6. We are already considering other complexes for evaluation in future work. We used two bifacial solar cells from CENER (National Renewable Energy Centre) in Navarra, Spain. In one of the bifacial mini-modules (first experiment), we conducted a series of measurements when complex C6 is directly deposited on top of the mini-module by dissolving the complex in tetrahydrofuran (THF). A total of 300 microliters of the compound are deposited using a micropipette via drop-casting (DS film). For the second bifacial mini-module (second experiment), we encapsulated the compound deposited on glass with EVA (DS layer). In both cases, we performed the experiment with the light incident perpendicularly on both sides.

##### First Experiment

**Figure 22:**
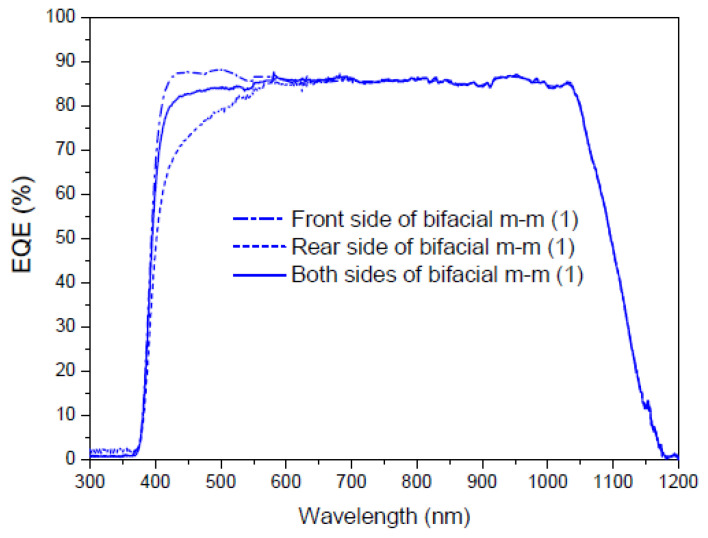
EQE values in percent of a bifacial mini-module, bm-m (1), when illuminated on the front (dash-dot line), rear (short dash line), and both sides (blue solid line) from 300 to 1200 nm.

Figure 22 shows the measurements obtained in the first experiment to obtain the EQE values of a multi-crystalline silicon bifacial mini-module in the spectral range of 300 to 1200 nm. The EQE dependence was obtained for the wavelength on the front side, on the rear side, and on both sides. The total EQE (illumination from both sides) is not the sum of the individual EQE*s* (illumination from each side), it is the sum of the EQE*s* of each side weighted by a factor that depends on the percentage of illumination from each side, where the sum of those factors is the total incident light.

**Figure 23:**
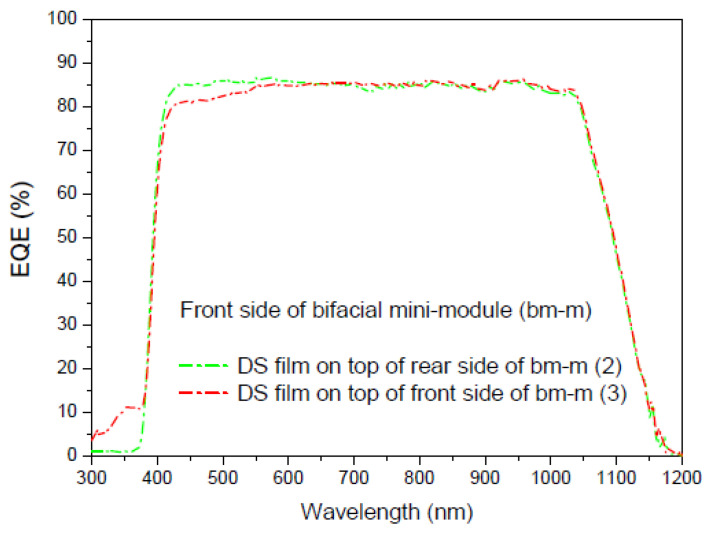
EQE values in percent of the bifacial mini-module with down-shifter films of complex C6 on top of the rear side (green line), bm-m (2), and both sides (red line), bm-m (3), when illuminated on the front side from 300 to 1200 nm.

**Figure 24:**
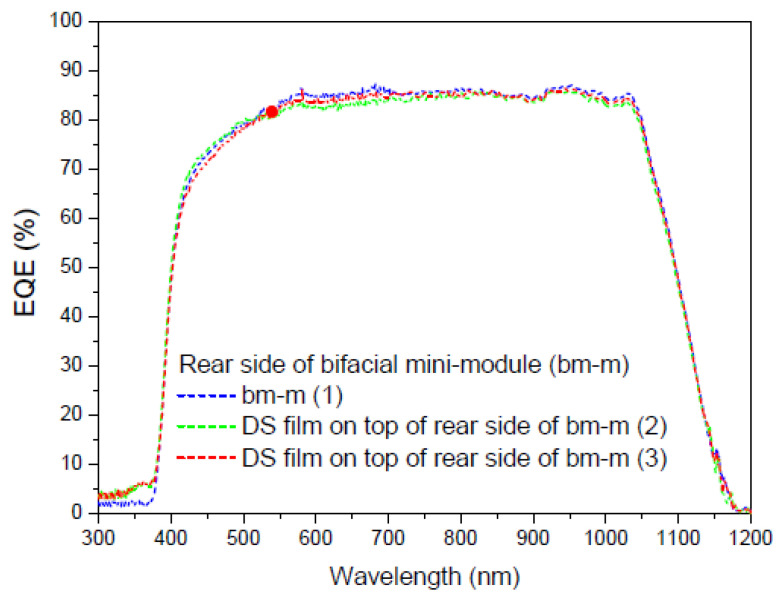
EQE values in percent of the bifacial mini-module with down-shifter films of complex C6 on top of the rear side (green line), bm-m (2), and both sides (red line), bm-m (3), when illuminated on the rear side from 300 to 1200 nm.

**Figure 25:**
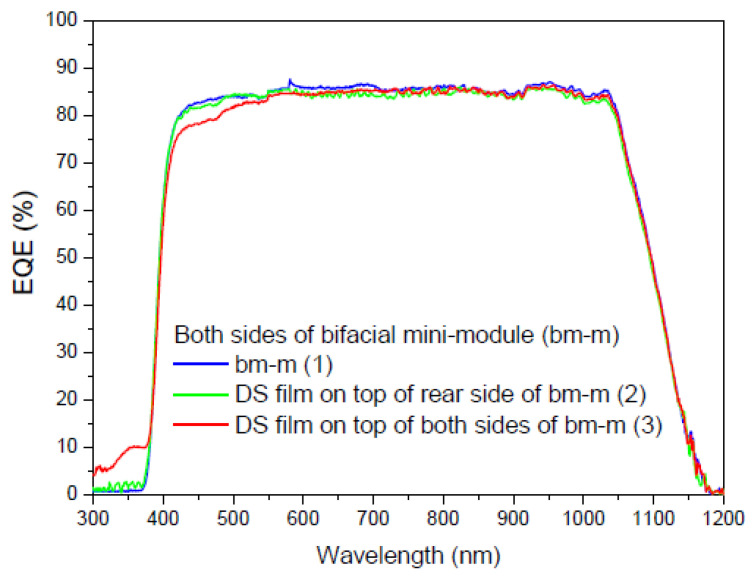
EQE values in percent of the bifacial mini-module with down-shifter films of complex C6 on top of the rear side (green line), bm-m (2), and both sides (red line), bm-m (3), when illuminated on both sides from 300 to 1200 nm.

Once we have deposited the DS film on the faces of the bifacial mini-module, we perform different measurements. The first measurement is taken when light illuminates the sample deposited on the front face of the mini-module, the second measurement is taken with the sample deposited on the rear face, and the third measurement is taken when light illuminates the sample on both faces simultaneously. As shown in Figure 22, the experimental setup requires the light beam to be split at the output of the monochromator using the necessary optics, including mirrors and lenses. Figure 23, Figure 24 and Figure 25 display the EQE results for the three cited cases.

Evaluation of the current density was performed using EQE measurements, and we calculated a parameter called the bifaciality factor, *BF* (%), which is defined as the ratio between the value of J*_sc_* when illuminated from the rear side and its value when illuminated solely from the other side [82]. Table 7 presents the calculated values for all EQE measurements conducted (Figure 23 and Figure 24).

As the EQE cannot be used to assess current values with illumination from both sides of the bifacial cell (Figure 24), the main conclusion here is that, in addition to achieving an increase in the bifaciality factor when the film is placed on the rear side (+20.6%) in the UV-vis range, there is an overall increase in the bifaciality factor across the entire spectral range when films are placed on both sides (0.76%), even though some loss occurs in the UV-vis range. These conditions are considered non-field working conditions. Light at different incidences should be studied in future works.

##### Second Experiment

**Figure 26:**
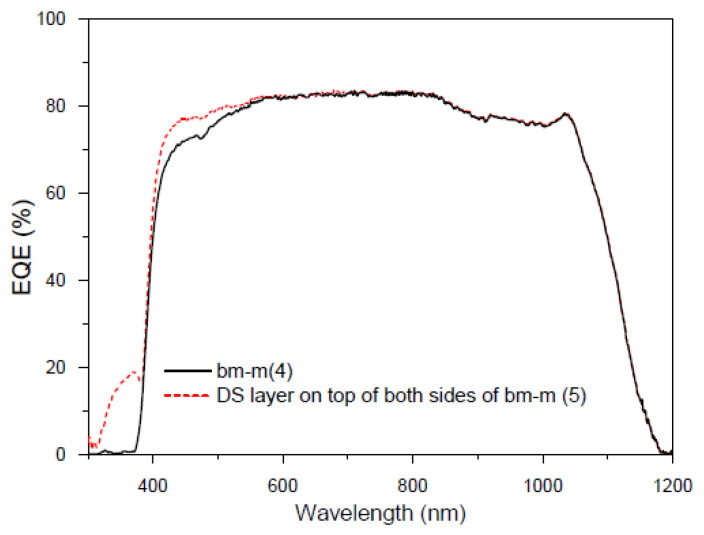
EQE values in percent of the bifacial mini-module bm-m(4) (black line), and when a DS layer of complex C6 is encapsulated on both sides bm-m(5) (red line) when illuminated on both sides from 300 to 1200 nm.

In this second experiment, we have encapsulated the compound used in the first experiment with EVA on another bifacial mini-module. We present preliminary EQE results in Figure 26, measuring with normal incidence from both sides simultaneously, bm-m(4), and repeating the measurement when the DS layer is encapsulated on both sides, bm-m(5).

When the increase in EQE obtained by encapsulating the DS film onto the mini-module was observed, we decided to conduct some experiments measuring the IV curves as detailed below. As mentioned earlier, it is not possible to extract a current value from the EQE measurement with illumination from both sides, as it would be a linear combination of the currents measured on each side rather than their sum.

**Figure 27:**
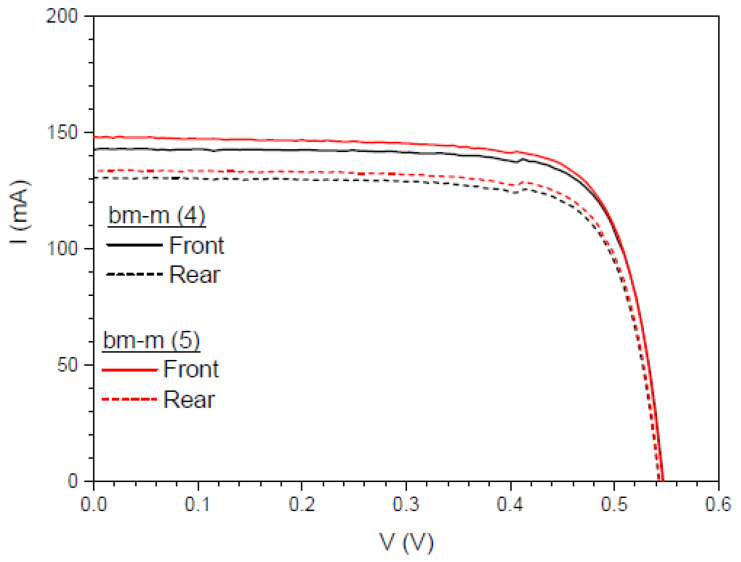
IV curves of the bifacial mini-module, bm-m(4) (black lines), and when a DS layer of complex C6 is encapsulated on both sides, bm-m(5) (red lines), when illuminated on the front side (solid lines) and the rear side (dashed lines), all under one Sun of illumination.

In Figure 27, we present IV measurements with one Sun of illumination first on the front side and then on the rear side, considering an effective area of 4 cm^2^. The Table 8 presents the calculated values of the IV measurements shown in Figure 27.

**Figure 28:**
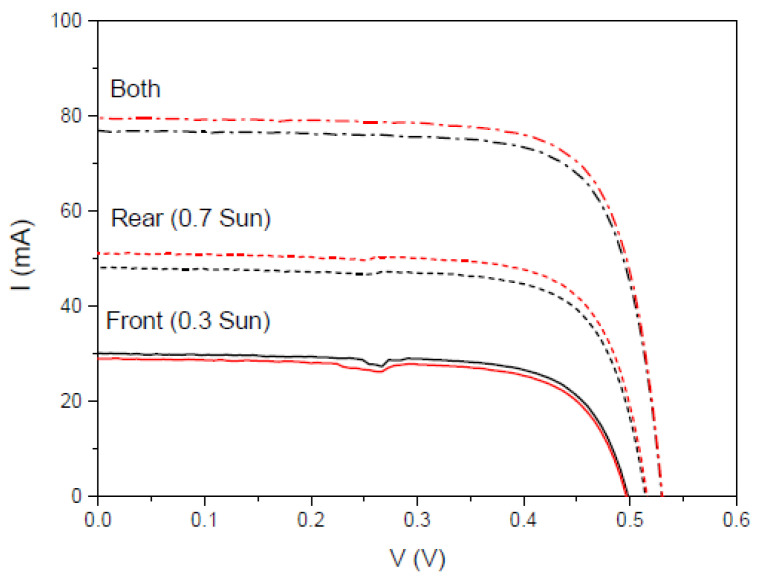
IV curves of the bifacial mini-module, bm-m(4) (black lines), and when a DS layer of complex C6 is encapsulated on both sides, bm-m(5) (red lines), when illuminated on the front side under 0.3 Sun of illumination (solid lines), on the rear side under 0.7 Sun of illumination (short dash lines), and when illuminating both sides (dash-dot lines).

We also calculated another parameter called the separation rate, *SR* (%), which is intended to quantitatively assess the difference between the sum of J*_sc_* when illuminated from each side of the bifacial cell and J*_sc_* when illuminated simultaneously from both sides [83]. We have studied the separation rate in a more realistic situation where the fraction of incident light from one side of the bifacial cell is lower than from the other side. In Figure 28, we present IV measurements for an illumination of 0.7 Sun on the rear side and 0.3 Sun on the front side. Table 9 presents the calculated values of the IV measurements shown in Figure 28.

Just as the bifaciality factor gives the percentage of photogenerated current between the rear and front of the mini-module, the separation rate gives the percentage of current that separates the measured current when both sides are illuminated using the sum of the photogenerated currents of the front and rear sides of the mini-module.

## 4. Conclusions

This work presents the results of our most recent research on the synthesis, characterization, and stability of luminescent complexes as down-shifters for their application in improving the efficiency of photovoltaic solar cells. The results and applications of three series of photoluminescent complexes as down-shifters are given.

For the first series of complexes, the strengths of complexes C1–3 and C6 as down-shifters are summarized, and new complexes C4 and C5 were characterized as potential candidates as well. The first studies of complexes C1 and C2 to create DS layers were carried out and an increase in efficiency conversion of 0.1% was obtained. DS layers were studied for different concentrations of the complex C3, and around 30% was found to be the limit of concentration. For PV mini-modules based on a single p-type mc-Si solar cell with a 16% conversion efficiency, the increases were 0.17% and 0.31%, for 10% and 30% concentrations of complex C3, respectively. The increase was 0.46% for 30% C3 when a hemispherical reflector (concentrator) was placed on top of the down-shifter.

Different experimental configurations of encapsulated DS layers of complex C6 were studied when they were exposed to UV aging to find the optimal configuration.

For the second series of complexes, possible applications of complexes C7–11 are highlighted through their photoluminescent characterization, and although they were not studied as DS layers, they are candidates for future work. The intense emission exhibited by the Er(III) and Yb(III) complexes suggests their potential applications in optical amplifiers and bio-imaging.

In our most recent investigations, we highlight the effectiveness of complexes C12–17 as down-shifters, particularly when utilizing bridging ligands like benzoate to form heterodinuclear complexes. These films can be applied to encapsulate a photovoltaic multi-crystalline silicon mini-module, resulting in improved efficiency through the down-shifting process for all the compounds within the 300–400 nm range. Additionally, the DS layers of complexes C14 and C17 demonstrate enhanced performance across the 300–1200 nm spectral range.

We have also applied complex C6 in a study of the use of DS layers in a bifacial cell, obtaining preliminary results that may be interesting for these types of devices.

## Figures and Tables

**Table 1:** Molecular structures of complexes C1–6.

Complex	Molecular Structure	Schematic View
[Eu_2_(phen)_2_(bz)_6_] (C1)	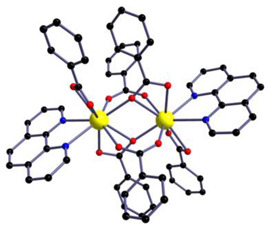	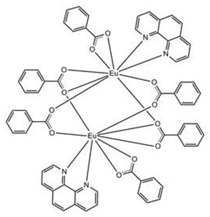
[Eu_2_(bphen)_2_(bz)_6_] (C2)	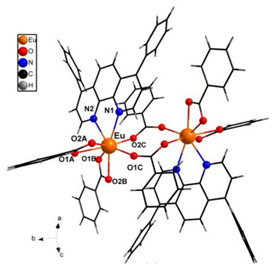	(*)
[Eu(tta)_3_bphen] (C3)	(**)	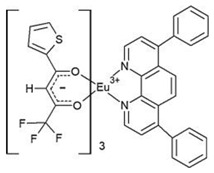
[Eu(bta)_3_pyz-phen] (C4)	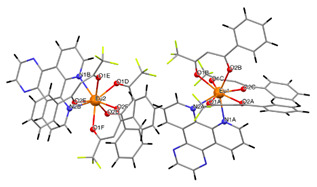	(***)
[Eu(tta)_3_pyz-phen] (C5)	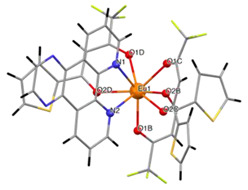	
[Eu(bta)_3_(me-phen] (C6)	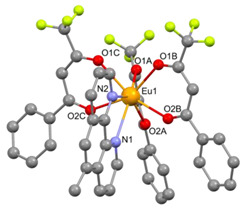	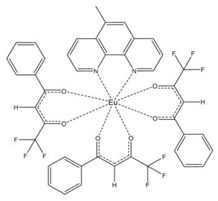

(*) Analogous to the complex (C1), the phen and bphen ligands are being exchanged; (**) see reference [70] for the crystal structure of the complex Eu(tta)_3_phen. Complex C3 has been characterized using other techniques in previous studies [26,27,28,29]; however, to the best of our knowledge, there are no data on the structural resolution of this complex; (***) analogous to the complex (C6), the me-phen and pyz-phen ligands are being exchanged.

**Table 2:** Coordination environment of Yb(III) and Er(III) and molecular structures of complexes C7–11.

Complex	Molecular Structure
[Yb(bta)_3_(me-phen)] (C8) (*)	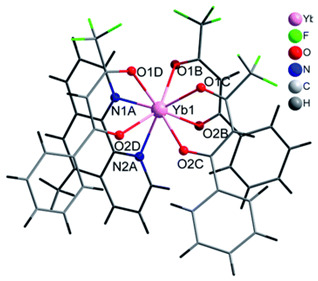
[Yb(bta)_3_(pyz-phen)] (C10)	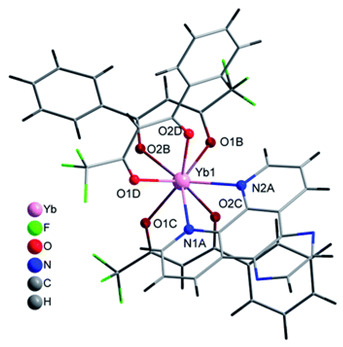
[Er(tta)_3_(pyz-phen)] (C11)	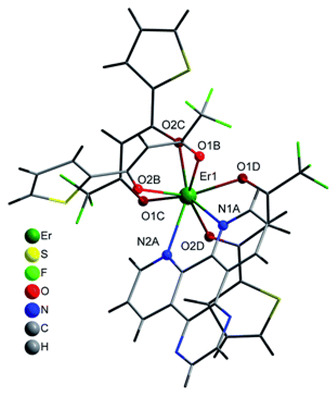

(*) C8 was found to be isostructural with the corresponding Er(III) and Gd(III) complexes (C7 and C9, respectively).

**Table 3:** Molecular structures of complexes C12–17 [M_2_(bz)_4_(tta)_2_(phen)_2_], where M_2_ metal- ions can be Eu_2_, Gd_2_, EuTb, or EuGd.

Complex	Molecular Structure
[Eu_2_(bz)_4_(tta)_2_(phen)_2_] (C12) (*)	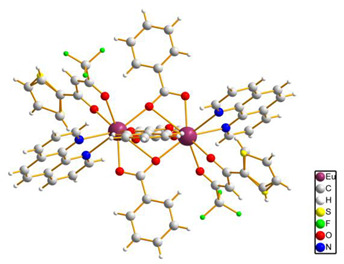

(*) C12 was found to be isostructural with the corresponding complexes C13–17.

**Table 4:** Lifetime (τ) and PLQY of the complexes.

Complex	*λ_exc_* (nm)	*λ_em_* (nm)	*τ* (ms)	PLQY (%)	Reference
C1	340	614	1.02	48	[25]
C2	280		0.96	50	this work, [79]
C3	340	612	0.98	69	this work, [26,80]
C4	375		0.53		this work
C5	375		0.81		
C6	350		0.54		
C7	375	1550	1.6 × 10^−3^		this work, [32]
C8		980	7.2 × 10^−3^		
C10		980	6.1 × 10^−3^		
C11		1550	1.3 × 10^−3^		
C12	350	612	0.60	63	this work, [33]
C14			0.75	66	
C15			0.65	50	
C16			0.72	70	
C17			0.74	74	

**Table 5:** Calculated values of the increment in relative percentage of conversion efficiency, (Δ*η*/*η*) % for C1 complex at 2% of concentration.

Complex Used	(Δ*η*/*η*) (%) (285–350 nm)	(Δ*η*/*η*) (%) (285–1200 nm)
C1 (tape-cast)	279.0	0.1
C1 (spin-coated)	143.0	<0.1
C1 (tape-cast and reflector)	347.0	0.18

**Table 6:** Increase in EQE values at 370 nm (ΔEQE), and J*_sc_* values for the 300–1200 nm spectral range and for the 300–400 nm spectral range of the mini-module (m-m) with the DS layers encapsulated, as well as their increments (ΔJ*_sc_*) compared to the m-m for the 300–1200 nm spectral range and the 300–400 nm spectral range when there is no complex. The estimates for the increase in relative percentage of conversion efficiency (Δ*η*/*η*) % compared to the m-m when there is no complex are also displayed.

Complex	ΔEQE (%) ^a^	J*_sc_* (mA/cm^2^) ^b^		ΔJ*_sc_* (mA/cm^2^) ^b^	(Δ*η*/*η*) (%) ^b^	J*_sc_* (mA/cm^2^) ^c^	ΔJ*_sc_* (mA/cm^2^) ^c^	(Δ*η*/*η*) (%) ^c^
m-m used for C6	—	28.40				0.18	—	
C6	10.0	28.70		0.30	1.05	0.29	0.11	61.1
m-m used for C12–17	—	^d^ 25.80	^e^ 26.86			0.05	—	
C12	9.7	25.53	26.46	—	—	0.13	0.08	160
C13	11.4	24.79	25.60	—	—	0.15	0.10	200
C14	16.3	26.69	26.95	0.89; 0.09	3.45; 0.33	0.20	0.15	300
C16	10.9	25.40	25.78	—	—	0.14	0.09	180
C17	14.3	26.04	27.04	0.24; 0.18	0.93; 0.67	0.17	0.12	240

^a^ At 370 nm. ^b^ For the 300–1200 nm spectral range. ^c^ For the 300–400 nm spectral range. ^d^ J*_sc_* obtained from Intensity–Voltage (IV) curves with reflector [31]. ^e^ J*sc* obtained from EQE measurements.

**Table 7:** Calculated values of the current density, J*_sc_*, and bifaciality factor, *BF* for (a) 300–1200 nm and (b) 300–400 nm spectral ranges.

Device	J_*sc*_ (mA/cm^2^) Front	J_*sc*_ (mA/cm^2^) Rear	Bifaciality Factor (%)
(a) bm-m(1)	36.18	35.15	97.15
(b) bm-m(1)	19.00	14.49	76.26
(a) bm-m(2)	35.71	34.69	97.14
(b) bm-m(2)	19.56	17.99	91.97
(a) bm-m(3)	35.60	34.85	97.89
(b) bm-m(3)	25.04	17.16	68.53

**Table 8:** Calculated values of the short circuit current *I_sc_*, bifaciality factor *BF*, maximum power *P_max_*, and the efficiency *η* for one Sun of illumination and a 4 cm^2^ area.

Device	*I_sc_* (mA) Front	*I_sc_* (mA) Rear	*BF* (%)	*P_max_* (mW) Front	*P_max_* (mW) Rear	*η* (%) Front	*η* (%) Rear
bm-m(4)	142.96	130.58	91.34	60.22	54.37	15.05	13.59
bm-m(5)	148.18	133.80	90.29	61.39	55.66	15.35	13.91

**Table 9:** Calculated values of the short circuit current *I_sc_*, the separate rate *SR*, and the maximum power *P_max_*, for 0.3 and 0.7 Sun of illumination on front and rear sides, respectively, and a 4 cm^2^ area.

Device	*I_sc_* (mA) Front	*I_sc_* (mA) Rear	*I_sc_* (mA) Both	*SR* (%)	*P_max_* (mW) Front	*P_max_* (mW) Rear	*P_max_* (mW) Both
bm-m(4)	30.04	48.09	76.85	−1.6	10.66	18.20	30.66
bm-m(5)	28.95	51.13	79.53	−0.7	10.16	19.46	31.77

## Supplementary Materials

The following supporting information can be downloaded at: https://www.mdpi.com/article/10.3390/ma16145068/s1, Figures S1–S7: Simulated and experimental X-ray powder diffraction patterns for the compounds. Figures S8–S11: Scheme of the energy transfer mechanism and photoluminescence process for the compounds. Table S1: Energies of the first excited singlet (ES_1_) and triplet (ET_1_) states in cm^−1^ with respect to their corresponding HOMO for the ligands used, and the energy gap Δε_ST_ in eV between the S_1_ and T_1_ states.
