# Supplementary material for: Exploring Ln(III)-Ion-Based Luminescent Species as Down-Shifters for Photovoltaic Solar Cells
Source: Materials (Basel). 2023 Jul 18;16(14):5068. doi: 10.3390/ma16145068 (PMC10383640; doi:10.3390/ma16145068)
Supplement: Supplementary file 1 [file materials-16-05068-s001.zip › Supplemantary Materials Figures S1-S7.pdf]

Supplementary Materials

Exploring Ln(III)-Ion-Based Luminescent Species as Down-Shifters for Photovoltaic Solar Cells

Gabriela Brito-Santos [1], Cecilio Hernández-Rodríguez [2,*], Beatriz Gil-Hernández [3], Joaquín Sanchiz [3], Inocencio R. Martín [2], Benjamín González-Díaz [4] and Ricardo Guerrero-Lemus [2]

[1] Departamento de Química, Facultad de Ciencias, Universidad de La Laguna (ULL), Avenida Astrofísico Francisco Sánchez S/N, 38206 La Laguna, Tenerife, Spain; gbritosa@ull.edu.es

[2] Departamento de Física, Facultad de Ciencias, Instituto Universitario de Materiales y Nanotecnología, Universidad de La Laguna (ULL), Avenida Astrofísico Francisco Sánchez S/N, 38206 La Laguna, Tenerife, Spain; imartin@ull.edu.es (I.R.M.); rglemus@ull.edu.es (R.G.-L.)

[3] Departamento de Química, Facultad de Ciencias, Instituto Universitario de Materiales y Nanotecnología, Universidad de La Laguna (ULL), Avenida Astrofísico Francisco Sánchez S/N, 38206 La Laguna, Tenerife, Spain; beagher@ull.edu.es (B.G.-H.); jsanchiz@ull.edu.es (J.S.)

[4] Departamento de Ingeniería Industrial, Escuela Superior de Ingeniería y Tecnología, Universidad de La Laguna, Camino San Francisco de Paula S/N, 38206 La Laguna, Tenerife, Spain; bgdiaz@ull.edu.es

* Correspondence: chdezr@ull.edu.es; Tel.: +34-922318243

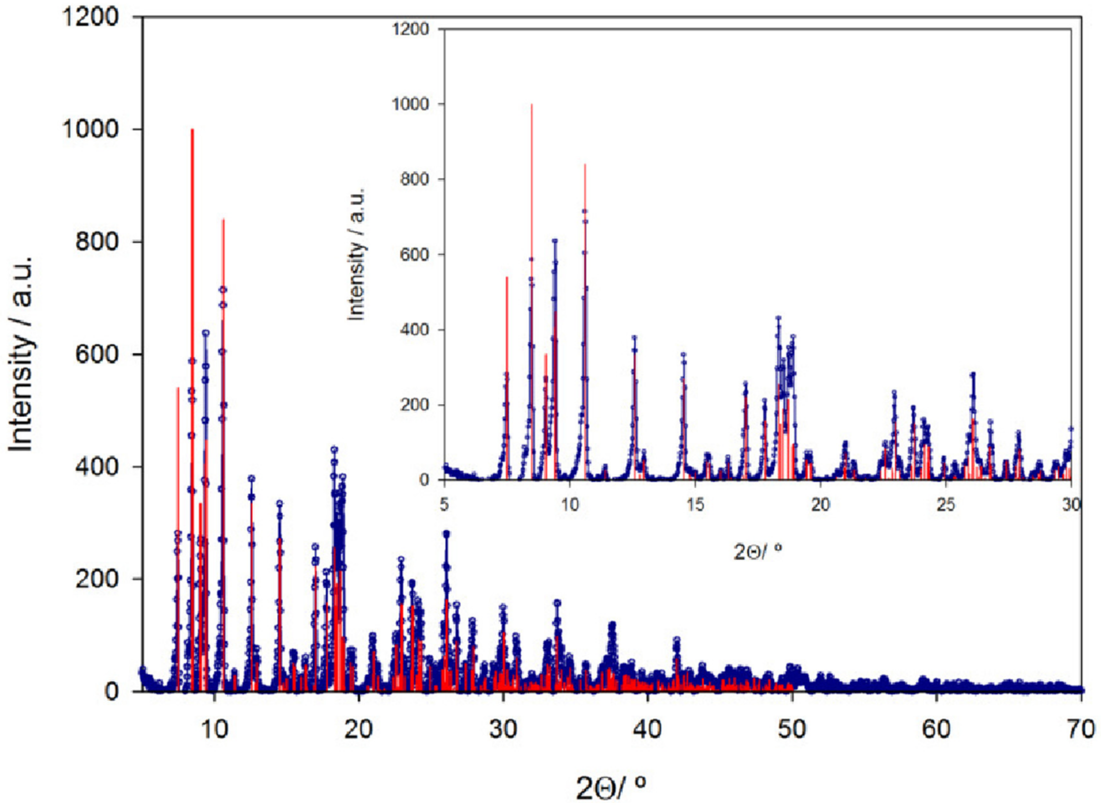

Figure S1. Simulated (red bars) and experimental (blue dots) X-Ray powder diffraction patterns for $[Eu_2(phen)_2(bz)_6]$.

Niu, S. Y.; Yang, B.; Cao, J. Q.; Yang, G. D.; Bu, W. M., Synthesis, characterization and structure of tetrabenzoyloxy bridged binuclear Eu(III) complex. Kao Teng Hsueh Hsiao Hua Heush Hsueh Pao/ *Chemical Journal of Chinese Universities* **1997**; *18*, 1917-1920.

Ooi, P. H.: Teoh, S. G.; Yeap, C. S.; Fun, H. K. Tetra-μ-benzoato-κ4O: O′; κ3O: O, O′; κ3O, O′: O′-bis [(benzoato-κ2O, O′)(1, 10-phenanthroline-κ2N, N′) europium (III)] benzoic acid disolvate. *Acta Crystallographica Section E: Structure Reports Online* **2010**, 66(5), m597-m598. doi: 10.1107/S1600536810015229. CCDC: 1258413.

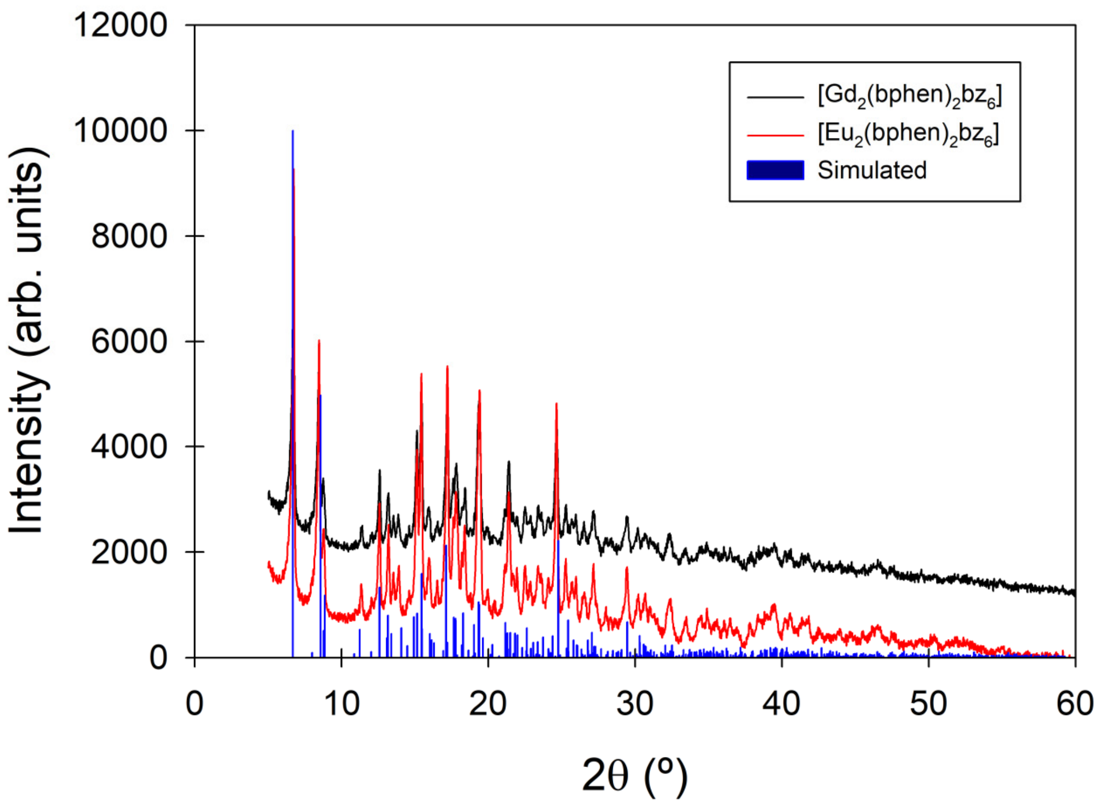

Figure S2. Experimental X-Ray powder diffraction patterns for compounds [Eu_2(bphen)$_2$(bz)$_6$] (red) and [Gd_2(bphen)$_2$(bz)$_6$] (black). There is an almost perfect overlap between both patterns which means both compounds display the same structure. The simulated spectrum from the single-crystal structure of [Eu_2(bphen)$_2$(bz)$_6$] is also showed (blue).

[25] González-Pérez, S.; Sanchiz, J.; Rodríguez, V. D.; Cañadillas-Ramallo, D.; González-Platas, J.; Borchert, D.; González-Díaz, B.; Hernández-Rodríguez, C.; Guerrero-Lemus, R. Highly luminescent film as enhancer of photovoltaic devices. *J. Lumin.* **2018**, *201*, 148–155. doi: 10.1016/j.jlumin.2018.04.038. CCDC: 1494543.

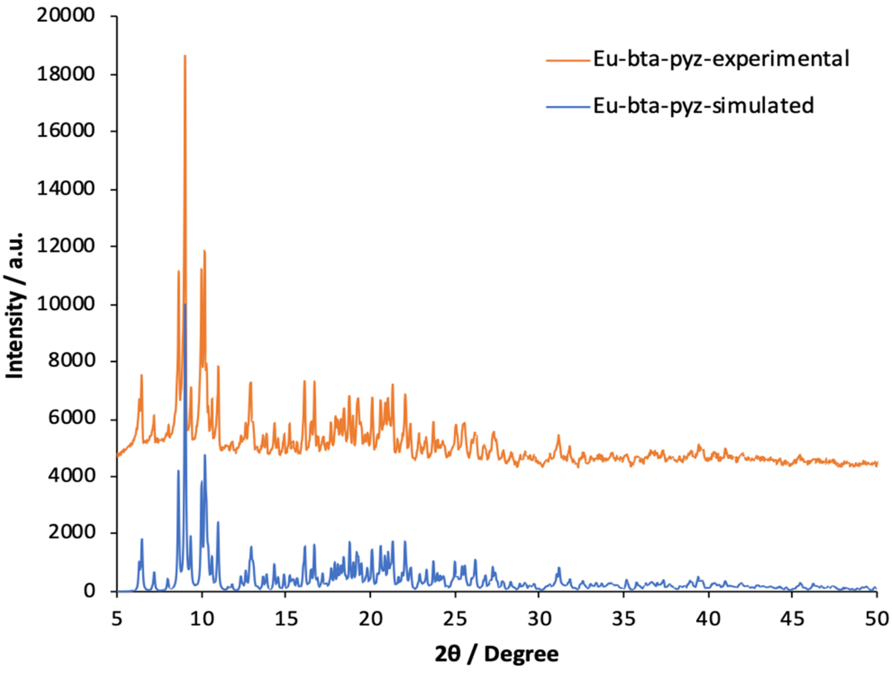

Figure S3.Simulated and experimental PXRD patterns (blue and orange lines, respectively for [Eu(bta)$_3$pyz-phen] complex.

Zhang, C.; Ma, X.; Cen, P.; Yang, H.; He, Z.; Guo, Y.; Liu, X. Dual-sensitized Eu (III)/Tb (III) complexes exhibiting tunable luminescence emission and their application in cellular-imaging. *Dalton Transactions* **2022**, 51(8), 3180-3187. doi: 10.1039/D2DT00051B. CCDC: 2030986.

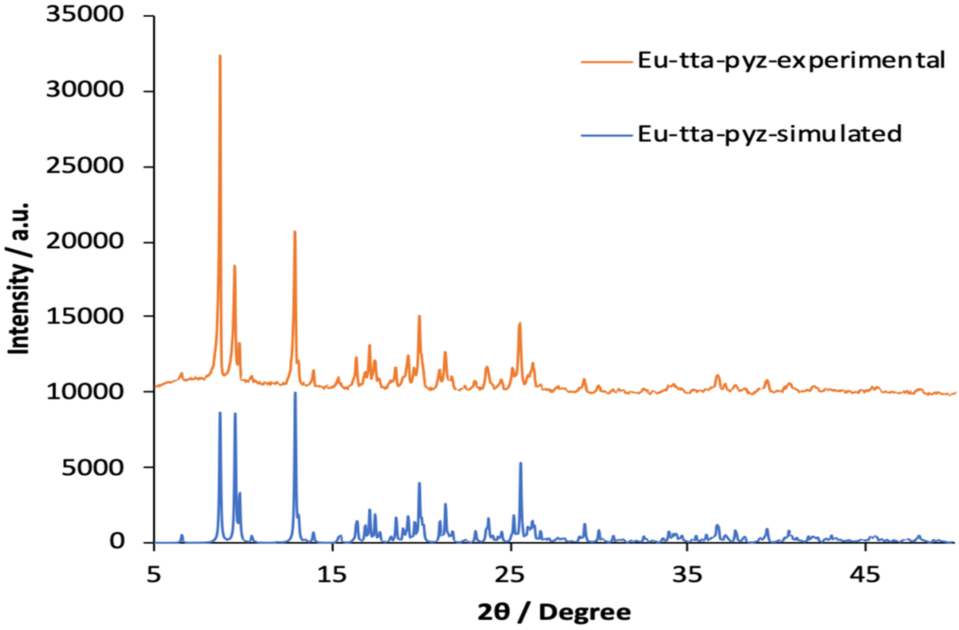

Figure S4.Simulated and experimental PXRD patterns (blue and orange lines, respectively for [Eu(tta)$_3$pyz-phen] complex.

Cabral, F. M.; Gálico, D. A.; Mazali, I. O.; Sigoli, F. A. Crystal structure and temperature dependence of the photophysical properties of the [Eu (tta) 3 (pyphen)] complex. *Inorganic Chemistry Communications* **2018**, 98, 29-33. doi: 10.1016/j.inoche.2018.09.041. CCDC: 1839940.

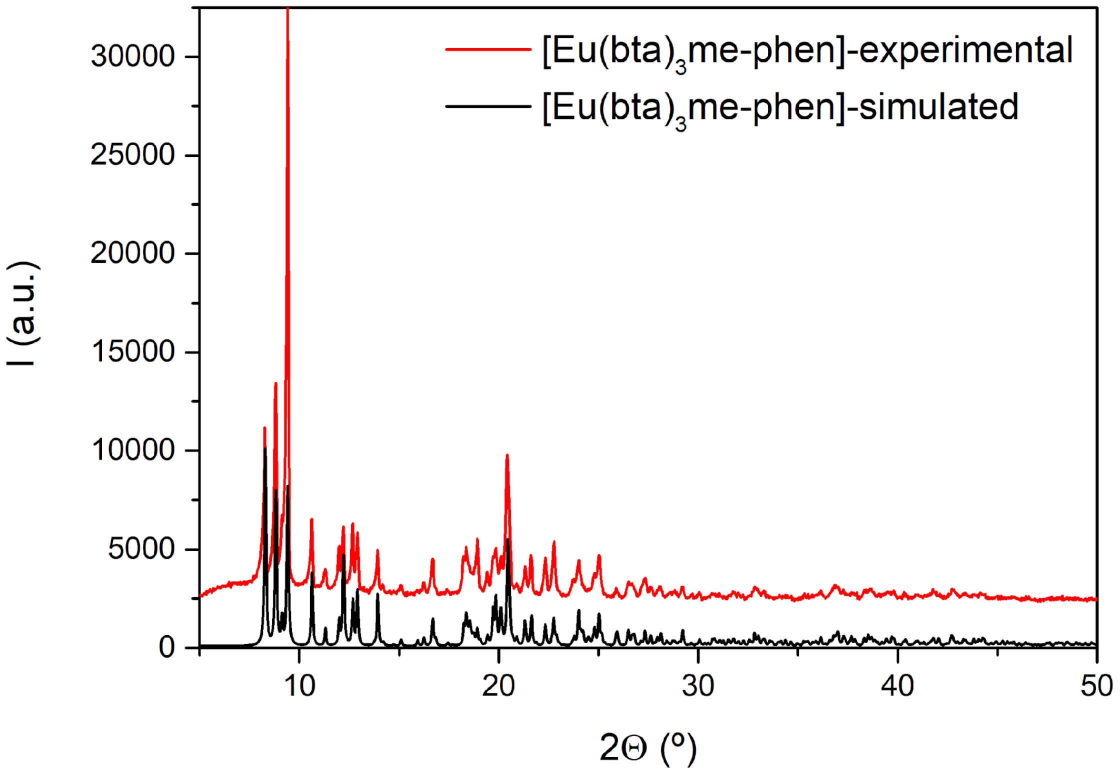

Figure S5.Simulated and experimental PXRD patterns (black and red lines, respectively for [Eu(bta)$_3$me-phen] complex.

[30] Brito-Santos, G.; Gil-Hernández, B.; Hernández-Rodríguez, C.; González-Díaz, B.; Guerrero-Lemus, R.; Sanchiz, J. Degradation analysis of highly UV-resistant down-shifting layers for silicon-based PV module applications. *Mater. Sci. Eng. B.* **2023**, *288*, 116207. doi: 10.1016/j.mseb.2022.116207. CCDC: 2169076.

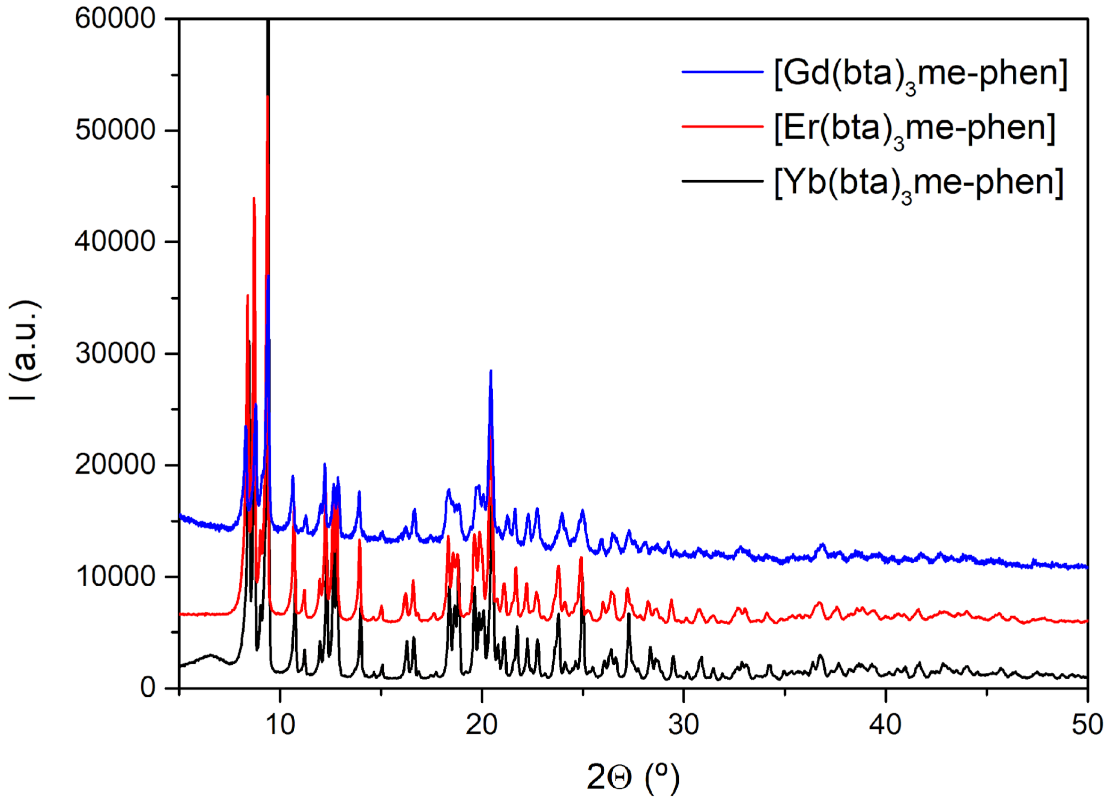

Figure S6. Experimental PXRD patterns of [Yb(bta)$_3$me-phen], [Er(bta)$_3$me-phen] and [Gd(bta)$_3$me-phen].

[30] Brito-Santos, G.; Gil-Hernández, B.; Hernández-Rodríguez, C.; González-Díaz, B.; Guerrero-Lemus, R.; Sanchiz, J. Degradation analysis of highly UV-resistant down-shifting layers for silicon-based PV module applications. *Mater. Sci. Eng. B*. **2023**, *288*, 116207. doi: 10.1016/j.mseb.2022.116207. CCDC: 2169076.

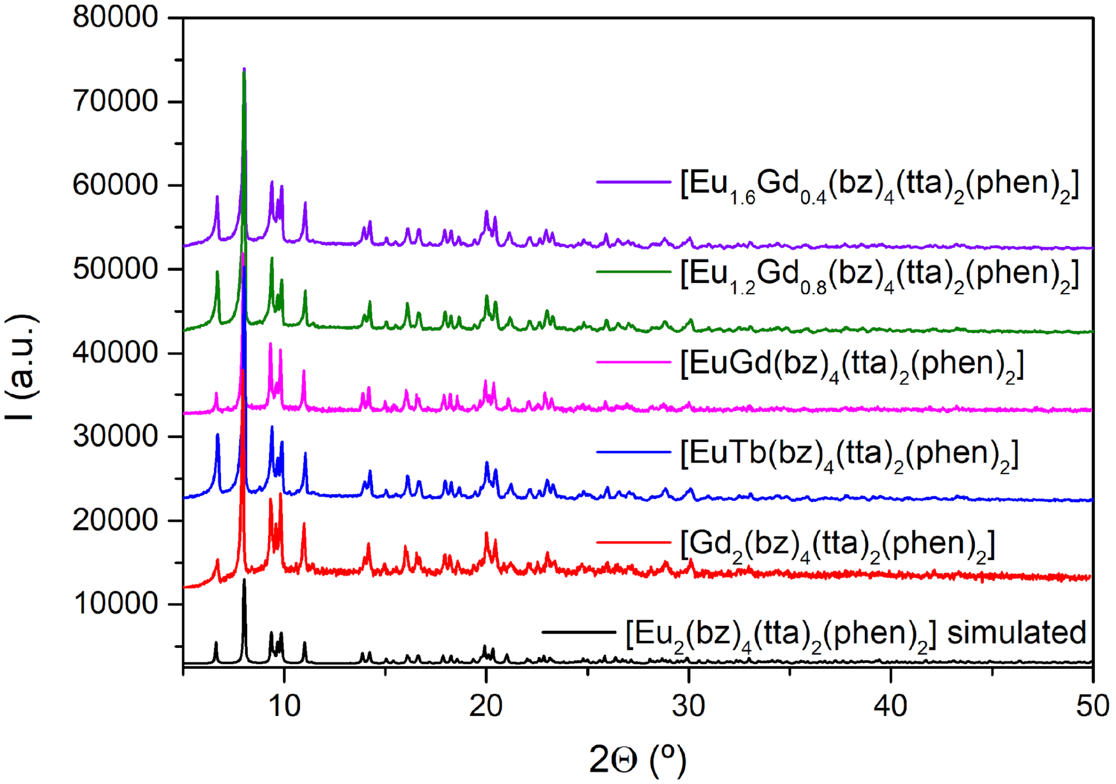

Figure S7. Experimental PXRD patterns of $[M_1M_2(bz)_4(tta)_2(phen)_2]$.

[33] Brito-Santos, G.; Hernández-Rodríguez, C.; Gil-Hernández, B.; González-Díaz, B.; Martín, I. R.; Guerrero-Lemus, R.; Sanchiz, J. Highly luminescent mixed-ligand bimetallic lanthanoid (III) complexes for photovoltaic applications. *Dalton Trans.* **2022**, *51(8)*, 3146–3158. doi: 10.1039/d1dt04248c. CCDC: 2110403.
