# Supplementary material for: Exploring Ln(III)-Ion-Based Luminescent Species as Down-Shifters for Photovoltaic Solar Cells
Source: Materials (Basel). 2023 Jul 18;16(14):5068. doi: 10.3390/ma16145068 (PMC10383640; doi:10.3390/ma16145068)
Supplement: Supplementary file 1 [file materials-16-05068-s001.zip › Supplemantary Materials Figures S8-S11.pdf]

**Supplementary Materials**

# Exploring Ln(III)-Ion-Based Luminescent Species as Down-Shifters for Photovoltaic Solar Cells


**Gabriela Brito-Santos [1], Cecilio Hernández-Rodríguez [2,*], Beatriz Gil-Hernández [3], Joaquín Sanchiz [3], Inocencio R. Martín [2], Benjamín González-Díaz [4] and Ricardo Guerrero-Lemus [2]**

1. Departamento de Química, Facultad de Ciencias, Universidad de La Laguna (ULL), Avenida Astrofísico Francisco Sánchez S/N, 38206 La Laguna, Tenerife, Spain; gbritosa@ull.edu.es
2. Departamento de Física, Facultad de Ciencias, Instituto Universitario de Materiales y Nanotecnología, Universidad de La Laguna (ULL), Avenida Astrofísico Francisco Sánchez S/N, 38206 La Laguna, Tenerife, Spain; imartin@ull.edu.es (I.R.M.); rglemus@ull.edu.es (R.G.-L.)
3. Departamento de Química, Facultad de Ciencias, Instituto Universitario de Materiales y Nanotecnología, Universidad de La Laguna (ULL), Avenida Astrofísico Francisco Sánchez S/N, 38206 La Laguna, Tenerife, Spain; beagher@ull.edu.es (B.G.-H.); jsanchiz@ull.edu.es (J.S.)
4. Departamento de Ingeniería Industrial, Escuela Superior de Ingeniería y Tecnología, Universidad de La Laguna, Camino San Francisco de Paula S/N, 38206 La Laguna, Tenerife, Spain; bgdiaz@ull.edu.es

* Correspondence: chdezr@ull.edu.es; Tel.: +34-922318243


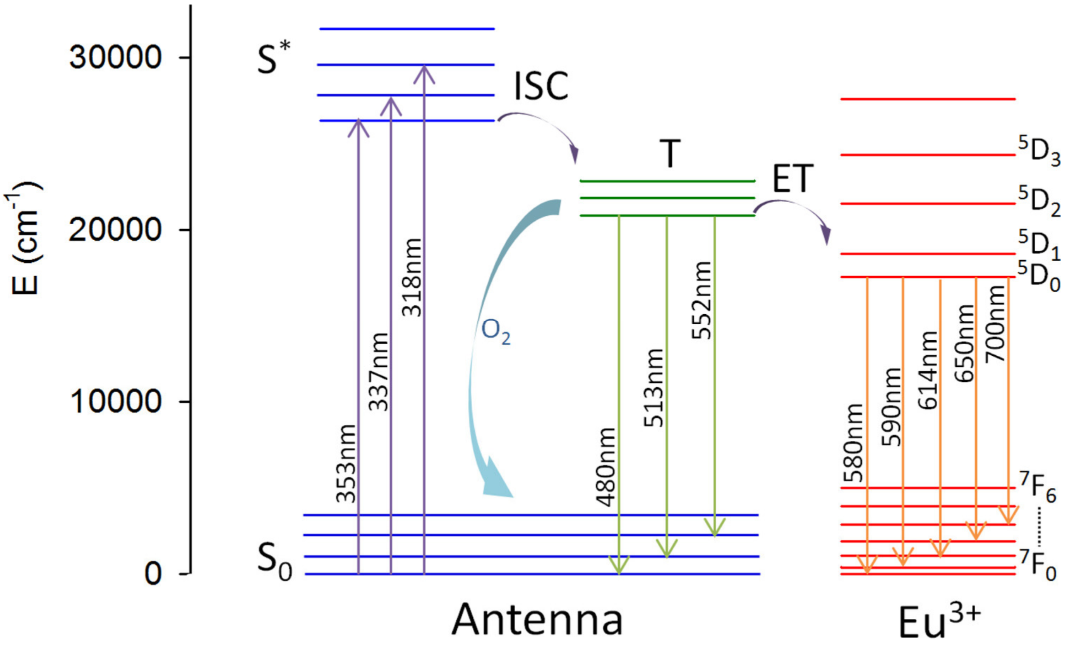


**Figure S8**. Photophysical processes in $[Eu_2(bphen)_2(bz)_6]$ under UV excitation taking to $Eu^{3+}$ red emission (antenna effect).

The diagram of Figure S8 shows the excitation of the antenna form the ground state $S_o$ to the excited S* (or $S_1$) single state, followed by intersystem crossing (ISC) to the triplet

state T, (or $T_1$). Subsequent quenching of the triplet states by molecular oxygen produces singlet oxygen ($O_2$). Finally, energy transference (ET) from the T state of the ligand to the excited state of the $Eu^{3+}$ ion is produced.

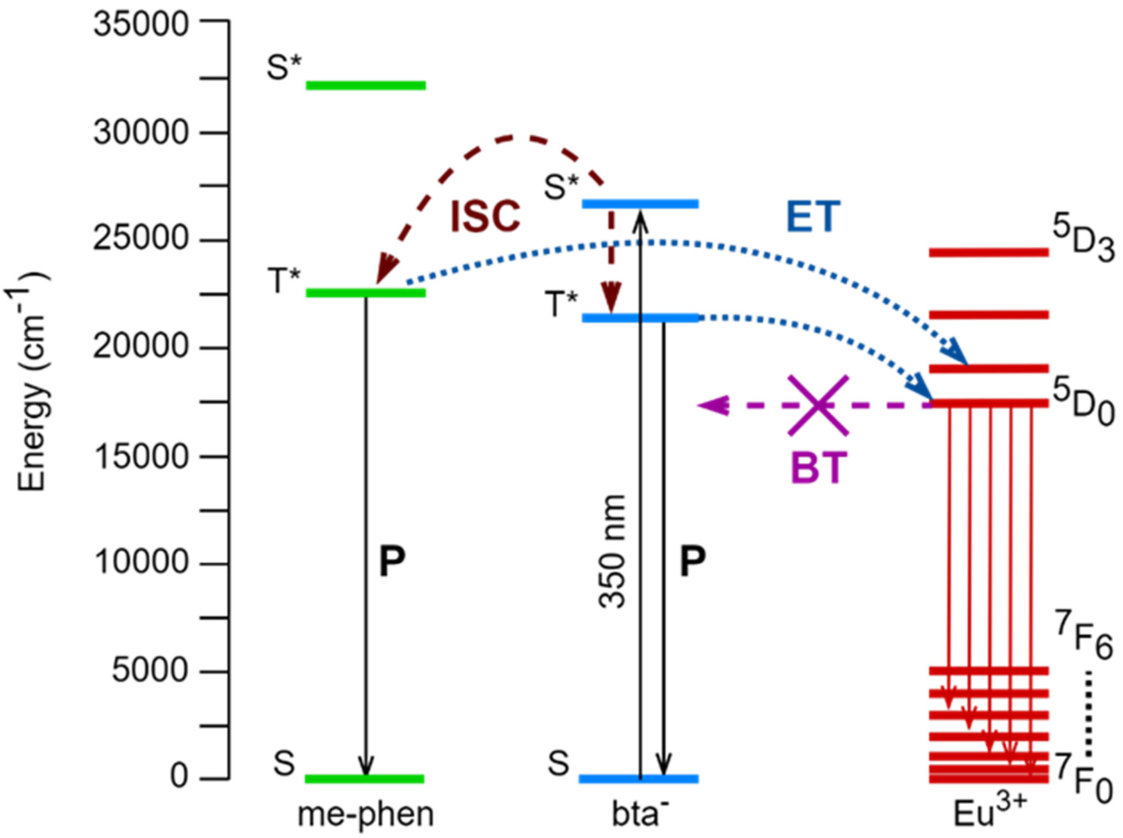


**Figure S9**. Figure 3.17. Scheme of the energy transfer mechanism and photoluminescence process for [Eu(bta)$_3$me-phen].

Energies of the states and the transitions that take place in the luminescent down-shifting process of [Eu(bta)$_3$me-phen] are shown in Figure S9.

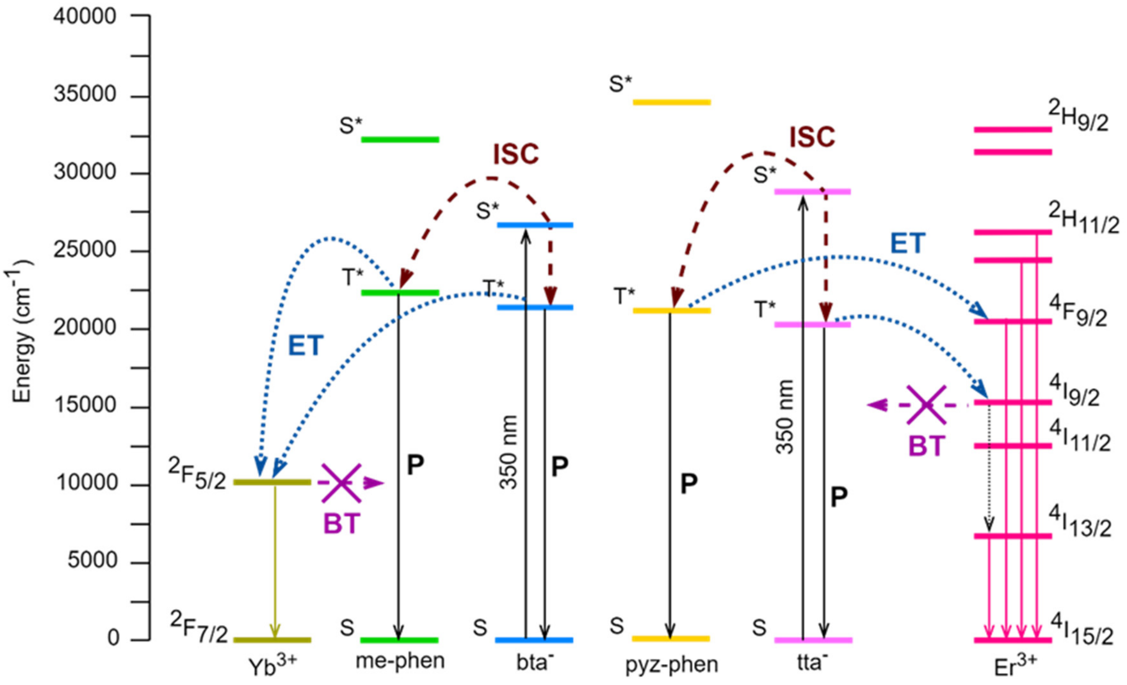


**Figure S10**. Scheme of the energy transfer mechanism and photoluminescence process for Yb(III) and Er(III) complexes.

Figure S10 shows the energy diagram for Yb(III) and Er(III) complexes with the β-diketonates bta- and tta- and the 1,10-phenanthroline derivates me-phen and pyz-phen.

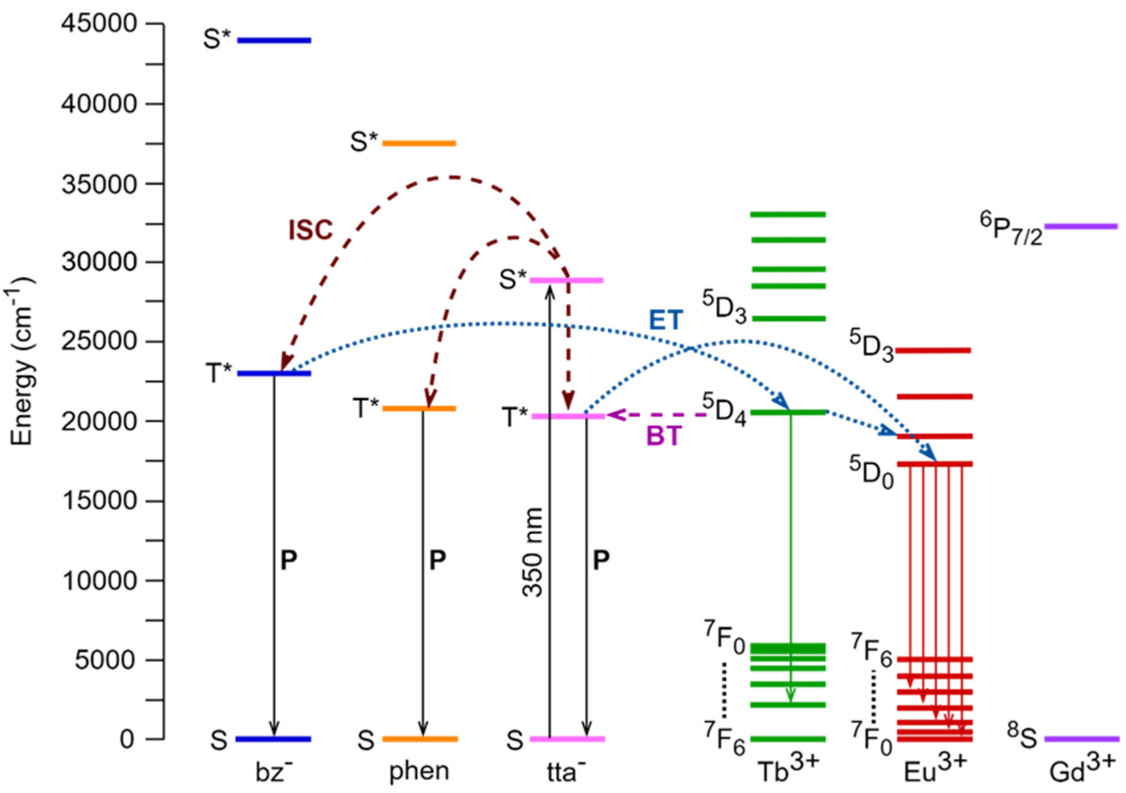


**Figure S11**. Scheme of the energy transfer mechanism and photoluminescence process for $[M1M2(bz)_4(tta)_2(phen)_2]$ series.

The energy diagram for $[M1M2(bz)_4(tta)_2(phen)_2]$ series is shown in figure S11.
