# Supplementary material for: Exploring Ln(III)-Ion-Based Luminescent Species as Down-Shifters for Photovoltaic Solar Cells
Source: Materials (Basel). 2023 Jul 18;16(14):5068. doi: 10.3390/ma16145068 (PMC10383640; doi:10.3390/ma16145068)
Supplement: Supplementary file 1 [file materials-16-05068-s001.zip › Supplemantary Materials Table S1.pdf]

Supplementary Materials

Exploring Ln(III)-Ion-Based Luminescent Species as Down-Shifters for Photovoltaic Solar Cells

Gabriela Brito-Santos [1], Cecilio Hernández-Rodríguez [2,*], Beatriz Gil-Hernández [3], Joaquín Sanchiz [3], Inocencio R. Martín [2], Benjamín González-Díaz [4] and Ricardo Guerrero-Lemus [2]

1 Departamento de Química, Facultad de Ciencias, Universidad de La Laguna (ULL), Avenida Astrofísico Francisco Sánchez S/N, 38206 La Laguna, Tenerife, Spain; gbritosa@ull.edu.es
2 Departamento de Física, Facultad de Ciencias, Instituto Universitario de Materiales y Nanotecnología, Universidad de La Laguna (ULL), Avenida Astrofísico Francisco Sánchez S/N, 38206 La Laguna, Tenerife, Spain; imartin@ull.edu.es (I.R.M.); rglemus@ull.edu.es (R.G.-L.)
3 Departamento de Química, Facultad de Ciencias, Instituto Universitario de Materiales y Nanotecnología, Universidad de La Laguna (ULL), Avenida Astrofísico Francisco Sánchez S/N, 38206 La Laguna, Tenerife, Spain; beagher@ull.edu.es (B.G.-H.); jsanchiz@ull.edu.es (J.S.)
4 Departamento de Ingeniería Industrial, Escuela Superior de Ingeniería y Tecnología, Universidad de La Laguna, Camino San Francisco de Paula S/N, 38206 La Laguna, Tenerife, Spain; bgdiaz@ull.edu.es
* Correspondence: chdezr@ull.edu.es; Tel.: +34-922318243

Table TS1. Energies of the first excited singlet (ES_1) and triplet (ET_1) states in cm^{-1} with respect to their corresponding HOMO for the ligands used, and the energy gap $\Delta\varepsilon_{ST}$ in eV between the S_1 and T_1 states.

Ligands	ES_1 (cm^{-1})	ET_1 (cm^{-1})	$\Delta\varepsilon_{ST}$ (eV)	References
phen	31000	20790	1.27	[33], [*]
bphen	31447	18116	1.65	[25]
me-phen	32300	18400	1.72	[32]
pyz-phen	34500	21200	1.65	[*], [32]
tta-	28900	20450	1.04	[33]
bta-	31200	28488	0.34	[**]
bz-	44000	23000	2.60	[33]

Although the bz- ligand absorbs quite low in the UV range and, therefore, is not very suitable for the applications in our work on its own, it can be interesting when combined with other ligands.

References

[*] Sun, Q.; Yan, P.; Niu, W.; Chu, W.; Yao, X.; An, G.; Li, G. NIR luminescence of a series of benzoyl trifluoroacetone erbium complexes. *RSC Adv.*, **2015**, *5*, 65856-65861. doi: 10.1039/c5ra12954k.

[**] Zhang, C.; Ma, X.; Cen, P.; Yang, H.; He, Z.; Guo, Y.; Liu, X. Dual-sensitized Eu (III)/Tb (III) complexes exhibiting tunable luminescence emission and their application in cellular-imaging. *Dalton Trans*. **2022**, *51(8)*, 3180-3187. doi: 10.1039/D2DT00051B.
